# Searching for signatures across microbial communities: Metagenomic analysis of soil samples from mangrove and other ecosystems

**DOI:** 10.1038/s41598-017-09254-6

**Published:** 2017-08-18

**Authors:** Madangchanok Imchen, Ranjith Kumavath, Debmalya Barh, Vasco Azevedo, Preetam Ghosh, Marcus Viana, Alice R. Wattam

**Affiliations:** 1 Department of Genomic Science, School of Biological Sciences, Central University of Kerala, Periye, Padanakkad P.O, Kasaragod, Kerala 671314 India; 2 Centre for Genomics and Applied Gene Technology, Institute of Integrative Omics and Applied Biotechnology, Nonakuri, Purba Medinipur, West Bengal 721172 India; 3 Xcode Life Sciences, 3D Eldorado, 112 Nungambakkam High Road, Nungambakkam, Chennai, Tamil Nadu 600034 India; 4 Laboratório de Genética Celular e Molecular, Departamento de Biologia Geral, Instituto de Ciências Biológicas (ICB), Universidade Federal de Minas Gerais, Pampulha, Belo Horizonte, Minas Gerais Brazil; 5 Department of Computer Science, Virginia Commonwealth University, Richmond, Virginia 23284 USA; 6 Biocomplexity Institute, Virginia Tech University, Blacksburg, Virginia 24061 USA

## Abstract

In this study, we categorize the microbial community in mangrove sediment samples from four different locations within a vast mangrove system in Kerala, India. We compared this data to other samples taken from the other known mangrove data, a tropical rainforest, and ocean sediment. An examination of the microbial communities from a large mangrove forest that stretches across southwestern India showed strong similarities across the higher taxonomic levels. When ocean sediment and a single isolate from a tropical rain forest were included in the analysis, a strong pattern emerged with Bacteria from the phylum *Proteobacteria* being the prominent taxon among the forest samples. The ocean samples were predominantly Archaea, with *Euryarchaeota* as the dominant phylum. Principal component and functional analyses grouped the samples isolated from forests, including those from disparate mangrove forests and the tropical rain forest, from the ocean. Our findings show similar patterns in samples were isolated from forests, and these were distinct from the ocean sediment isolates. The taxonomic structure was maintained to the level of class, and functional analysis of the genes present also displayed these similarities. Our report for the first time shows the richness of microbial diversity in the Kerala coast and its differences with tropical rain forest and ocean microbiome.

## Introduction

The mangrove ecosystem plays a crucial role by acting as a buffer zone between land and sea, maintaining the sea level and protecting the coast^1^. Mangroves are a crucial component of the food chain in the saline coastal biome of the tropics and subtropics. Mangrove trees convert solar energy into organic matter via photosynthesis, with their leaves and branches serving as a source of energy and providing a habitat for a variety of aquatic organisms, which in turn, support a higher level in the food chain. This ecosystem is an enormous food web, supplying a myriad of microorganisms with both protection and nutrients^2,3^. It is considered to be one of the most critical in tropical regions, and also one of the most vulnerable to global climate change^4^.

The complexity of the mangrove microbial communities has generated deep interest among microbial ecologists. The dynamic environment of the mangrove ecosystem, brought about by the regular tidal variations, pH, temperature, salinity, light, rainfall and nutrient availability provides an excellent environment for a wide range of organisms with diversified functional roles^5^. Studies have shown that microbial communities play a vital role in this ecosystem, being essential for biogeochemical cycles and biocycling of most nutrients, including nitrogen^6,7^. Except for absorption of nutrients, transformation processes of nutrients can also be important for avoiding eutrophication^8^.

The impact that mangrove forests and their supporting ecosystem extends beyond their local ecological impact. Explorations of this ecosystem have revealed some interesting bioactive compounds that could have a potential impact on human health. These include some of the novel compounds with cytotxoic activity^9,10^, some p-aminoacetophenonic acids that may have antibiotic properties^11–13^, some indolesesquiterpenes that appear to have selective anti-HIV activity^14,15^ and an anti-fibrotic compound^9^.

Mangroves cover up to 152,000 km^2^ globally^8^, but their range is decreasing due to pollution, urbanization and other human activities. Conservation and research on mangroves are imperative due to their dominant role in the marine food chain and the recognition that they may play a role in human health by their potential ecological, industrial and pharmaceutical impacts^5^. There is an increasing urgency to understand the structural and functional architecture that underlies the mangrove ecosystem, and the microbial community is an important part that remains unexplored. With more than 98% of microbes currently unculturable^16^, examination of environmental samples by metagenomic analysis is now the only avenue available for exploring these communities. Until now, the few metagenomic studies in mangroves have been concentrated South America^17^. The South American mangrove ecosystems are important, but constitute only 11% of the range of mangroves across the globe^18^. In this study, we carried out the first detailed analysis of microbial communities within the mangrove ecosystems from four different locations in mangrove forests across Kerala, India, and compare them to a similar Brazilian ecosystem, a tropical rainforest, and samples taken from ocean sediment.

## Results

### Sequencing, quality control and annotation of proteins

A total of 64413433 sequencing reads were obtained from the four samples from Kerala with an average length of 254 ± 12.75 bases. Following quality trimming, a total of 54833910 (85.13% of the total) remained, including for 7809729 reads for PGD (average read length of 408 ± 138 bp), 7303470 reads for MAL (average read length of 408 ± 138 bp), 6414174 reads for VL1 (average read length of 200 ± 68 bp), and 5920270 reads for PYN (average read length of 193 ± 64 bp). A total of 18.6 GB (64 million reads) were obtained across all four of the Kerala mangrove samples, and 54 million reads (85.13%) were retained for further analysis following quality control. The sequences for these reads are publicly available from the MG-RAST server under the following IDs: 4671371, 4671370, 467136 and 4671368. Of the sequences that passed quality control, each had a certain percentage that mapped to proteins of known (PGD= 39%, MAL= 41.2%, VL1= 35.4%, and PYN= 35.4%) and also to proteins of unknown function (PGD= 7.5%, MAL= 41.2%, VL1= 44.5%, and PYN= 44.1%). Asymptotic rarefaction curves were generated for each sample indicating that the majority of taxonomic diversity was covered in the samples (data not shown).

### Taxonomic Diversity across the Kerala samples

**Figure 1:**
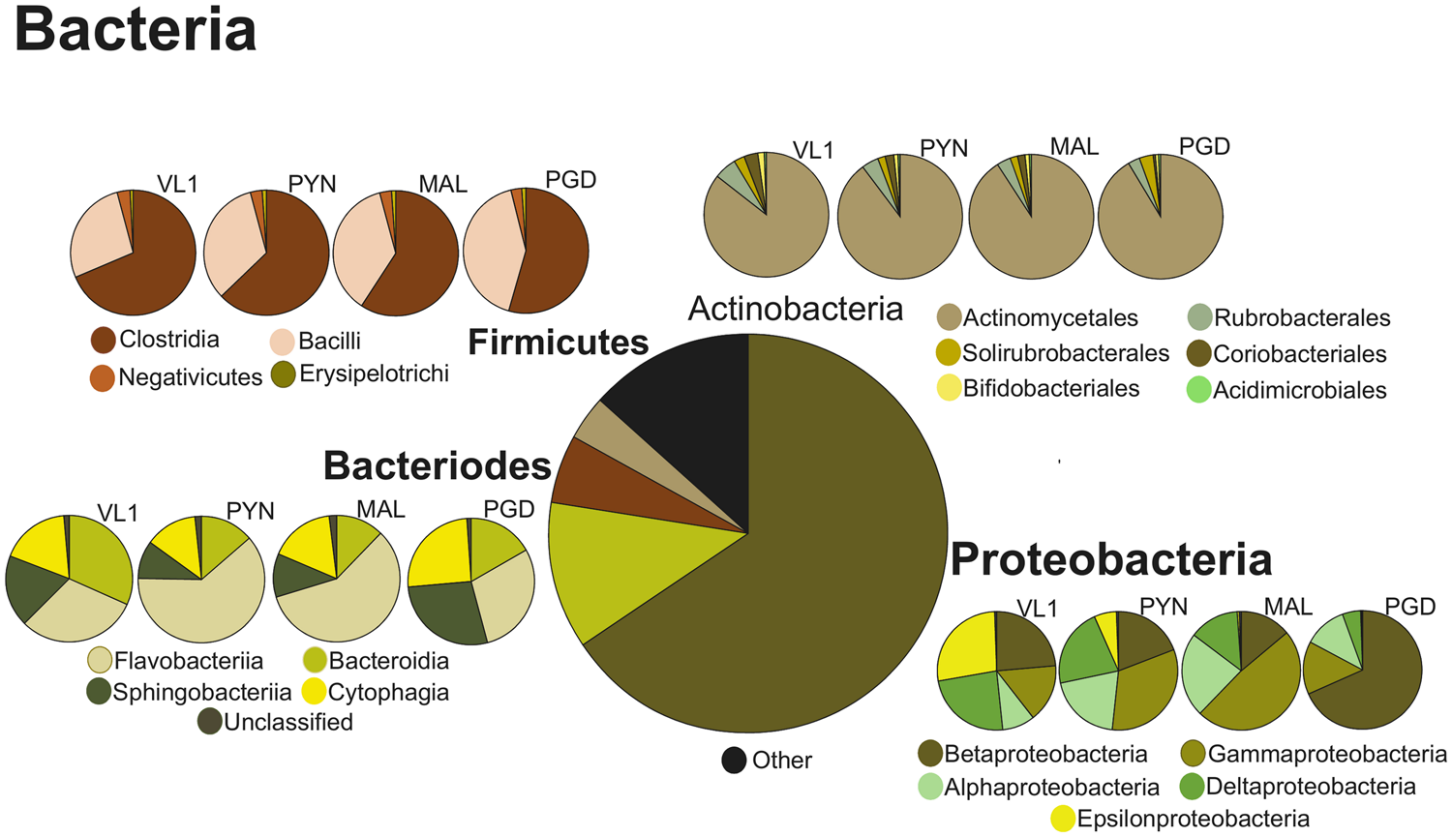
Taxonomic structure and diversity of the reads mapping to *Bacteria*. Kingdom, phyla and class divisions across the four sampling locations in Kerala, India, with class divisions noted across the top four phyla.

The taxonomic classifications of genes were assigned to the RefSeq annotation source^19^ using the Best Hit Classification algorithm of MG-RAST. A total of 13269130 representative sequences were assigned to different taxonomies using the RefSeq database from all the four datasets accounting for more than 99% of the reads for PYN, MAL, VL1 and PGD samples respectively (Table 1). There were also a number of sequences that could not be assigned to the highest taxonomy levels, with an average of 15.5% of the reads either unassigned, unclassified, or identified as other. Collectively, bacterial sequences dominated the overall reads accounting for 96.81% of the total assigned reads, with *Eukaryota* and *Archaea* assigned 1.8% and 1.32% respectively. Less than 1% of the reads mapped to viruses. Sequences from all the datasets were assigned to 28 different bacterial, 5 archaeal, and 34 eukaryotic phyla. All four samples shared *Proteobacteria* as the phyla with the most reads assigned, having an average of 65.70% (Supplementary Table 1). Similar rich dominance of *Proteobacteria* was found in Brazilian oil contaminated mangroves^20^. Other dominant bacterial phyla included *Bacteriodes* (11.83%), *Firmicutes* (5.56%) and A*ctinobacteria* (3.61%), but there was some variation in the ranking of each of these among the Kerala samples. Although all samples shared *Proteobacteria* as the predominant phylum, there was diversity in the percentage of the reads assigned the classes within that taxon (Fig. 1 and Table 2), with PYN and MAL both sharing *Gammaproteobacteria* as the dominant class, and VL1 and PGD with the majority of their *Proteobacteria* reads mapped to *Betaproteobacteria*. A similar lack of structure is seen within the phylum *Bacteriodes*. Both PYN and MAL have *Flavobacteriia* as the dominant class, but VL1 has most of its *Bacteriodes* reads map to *Bacteroidia*, and in PGD it is *Flavobacteria*. The classes within *Firmicutes* and *Actinobacteria* phyla have similar diversity and percentage of reads assigned to their top classes across all of the Kerala samples.

**Table 1:** Summary of reads and distribution across kingdoms in the samples from India, Brazil, Puerto Rico and from the isolates taken from the South China Sea.

**Sample Name**		**Kerala India Mangrove**						**Brazil Mangrove**						**South China Sea Ocean Sediment**						**Puerto Rico**
**MGRAST ID**		**PGD 4671368.3**	**MAL 4671369.3**	**PYN 4671370.3**	**VL1 4671371.3**	**Average**	**STD**	**BrMgv1 4451033.3**	**BrMgv2 4451034.3**	**BrMgv3 4451035.3**	**BrMgv4 4451036.3**	**Average**	**STD**	**E201-1 4487294.3**	**E201-2 4487295.3**	**E208-1 4487376.3**	**E208-2 4487377.3**	**Average**	**STD**	**Rain Forest 4446153.3**
**All Reads**	Total reads	3410117	3220906	3412553	3225554	3317283	108623.6	592698	107612	95675	96634	223154.8	246421.7	955453	977655	831502	710468	868769.5	123583.1	520500
	Total assigned	3410032	3220854	3412501	3225512	3317225	108611.4	592698	107610	95672	96632	223153	246422.8	955442	977611	831489	710415	868739.3	123591.5	520483
	Percent total assigned (%)	99.99750742	99.99839	99.99848	99.9987	99.99827	0.000523	100	99.99814	99.99686	99.99793	99.99823	0.001303	99.99884871	99.99549943	99.99843656	99.99254	99.99633	0.002935	99.99673
	Total unassigned	85	52	52	42	57.75	18.76832	0	2	3	2	1.75	1.258306	11	44	13	53	30.25	21.40677	17
	Percent total unassigned (%)	0.002492583	0.001614	0.001524	0.001302	0.001733	0.000523	0	0.001859	0.003136	0.00207	0.001766	0.001303	0.001151286	0.004500565	0.001563436	0.00746	0.003669	0.002935	0.003266
**Archaea**	Reads mapped to Archaea	16934	43985	59600	115670	59047.25	41660.69	13615	3946	2332	3469	5840.5	5227.031	120209	126753	44972	45395	84332.25	45284.29	6262
	Percent Archaea of total (%)	0.496581202	1.36561	1.746493	3.586051	1.798684	1.301336	2.297123	3.666877	2.437418	3.589834	2.997813	0.731014	12.58136193	12.96500299	5.408525776	6.38945	9.336085	3.992044	1.203074
	Percent Archaea of assigned (%)	0.49659358	1.365632	1.74652	3.586097	1.798711	1.301351	2.297123	3.666945	2.437495	3.589908	2.997868	0.731035	12.58150678	12.96558652	5.408610336	6.389927	9.336408	3.992115	1.203113
**Bacteria**	Reads mapped to Bacteria	3323940	3136206	3308800	3077852	3211700	123343.5	574258	102306	92059	91890	215128.3	239469.4	819037	834223	765291	647811	766590.5	84528.2	508545
	Percent Bacteria of total (%)	97.47290196	97.37031	96.95967	95.42088	96.80594	0.949626	96.8888	95.06932	96.22054	95.09075	95.81735	0.894065	85.72237462	85.32897597	92.03718091	91.18088	88.56735	3.533234	97.70317
	Percent Bacteria of assigned (%)	97.47533161	97.37188	96.96114	95.42212	96.80762	0.949982	96.8888	95.07109	96.22356	95.09272	95.81904	0.893494	85.72336154	85.33281643	92.03861987	91.18769	88.57062	3.533945	97.70636
**Eukaryota**	Reads mapped to Eukaryota	66029	39324	42955	28309	44154.25	15857.13	4726	1329	1238	1252	2136.25	1726.963	16004	16439	20596	16611	17412.5	2137.651	5455
	Percent Eukaryota total (%)	1.936267876	1.220899	1.258735	0.877648	1.323387	0.443091	0.797371	1.234992	1.293964	1.29561	1.155484	0.240402	1.675016981	1.681472503	2.476963375	2.338036	2.042872	0.424847	1.048031
	Percent Eukaryota assigned (%)	1.93631614	1.220918	1.258754	0.877659	1.323412	0.443107	0.797371	1.235015	1.294005	1.295637	1.155507	0.240417	1.675036266	1.681548182	2.477002101	2.338211	2.042949	0.424873	1.048065
**Virus**	Reads mapped to Virus	3129	1339	1146	3681	2323.75	1271.139	99	29	43	21	48	35.1947	192	196	630	598	404	242.8443	221
	Percent Virus of total (%)	0.091756383	0.041572	0.033582	0.11412	0.070258	0.038962	0.016703	0.026949	0.044944	0.021731	0.027582	0.012307	0.02009518	0.020047972	0.075766504	0.08417	0.05002	0.034751	0.042459
	Percent Virus of assigned (%)	0.09175867	0.041573	0.033582	0.114121	0.070259	0.038962	0.016703	0.026949	0.044945	0.021732	0.027582	0.012308	0.020095411	0.020048874	0.075767689	0.084176	0.050022	0.034753	0.042461
**Miscellaneous**	other sequences	85	52	52	42	57.75	18.76832	0	2	3	2	1.75	1.258306	11	44	13	53	30.25	21.40677	17
	unassigned (%)	0.002492583	0.001614	0.001524	0.001302	0.001733	0.000523	0	0.001859	0.003136	0.00207	0.001766	0.001303	0.001151286	0.004500565	0.001563436	0.00746	0.003669	0.002935	0.003266
	unclassified sequences (%)	0.002492645	0.001614	0.001524	0.001302	0.001733	0.000523	0	0.001859	0.003136	0.00207	0.001766	0.001303	0.0011513	0.004500768	0.00156346	0.00746	0.003669	0.002935	0.003266

**Table 2:** Class taxonomic distribution across the samples isolated from a mangrove forest in Kerala, India.

*Kingdom*	Phylum	*Bacteria* Class	VL1	PYN	MAL	PGD	Average	SD
*Bacteria*	*Proteobacteria*	*Gammaproteobacteria*	15.77	32.63	48.46	14.31	27.79	16.09
		*Betaproteobacteria*	23.80	19.20	14.04	68.59	31.41	25.11
		*Alphaproteobacteria*	8.94	20.07	22.92	11.56	15.87	6.68
		*Deltaproteobacteria*	23.83	21.49	13.52	5.09	15.98	8.50
		*Epsilonproteobacteria*	27.20	6.11	0.67	0.27	8.57	12.71
		*unclassified*	0.31	0.33	0.26	0.14	0.26	0.08
		*Zetaproteobacteria*	0.14	0.17	0.13	0.04	0.12	0.06
	*Bacteriodes*	*Bacteroidia*	31.93	13.83	12.35	16.92	18.76	8.99
		*Cytophagia*	17.64	13.44	16.74	25.12	18.23	4.93
		*Flavobacteria*	30.66	61.62	57.98	28.95	44.80	17.39
		*Sphingobacteria*	18.43	9.69	11.22	28.00	16.83	8.36
		*unclassified (derived from Bacteroidetes)*	1.34	1.42	1.72	1.02	1.37	0.29
	*Firmicutes*	*Bacilli*	27.22	33.08	36.63	41.67	34.65	6.08
		*Clostridia*	68.85	62.91	59.44	54.49	61.42	6.04
		*Erysipelotrichi*	0.74	0.84	0.85	0.87	0.83	0.06
		*Negativicutes*	3.19	3.17	3.09	2.96	3.10	0.10
	*Actinobacteria*	*Acidobacteria (class)*	8.73	6.53	6.99	7.82	7.52	0.97
		*Solibacteres*	22.03	17.59	15.46	12.59	16.92	3.98
		*unclassified (derived from Acidobacteria)*	11.65	7.97	7.87	9.68	9.29	1.78
		*Actinobacteria (class)*	57.58	67.91	69.68	69.92	66.27	5.86
*Archaea*	*Euryarchaeota*	*Archaeoglobi*	7.57	8.02	7.06	5.18	6.96	1.25
		*Halobacteria*	6.22	12.37	13.98	13.40	11.49	3.58
		*Methanobacteria*	7.29	6.93	6.30	6.35	6.72	0.48
		*Methanococci*	9.59	10.54	10.95	7.55	9.66	1.52
		*Methanomicrobia*	45.54	39.06	40.09	44.75	42.36	3.26
		*Methanopyri*	1.62	1.36	1.37	1.25	1.40	0.16
		*Thermococci*	12.31	13.41	12.45	10.87	12.26	1.05
		*Thermoplasmata*	2.34	2.16	2.22	3.27	2.50	0.52
		*unclassified (derived from Euryarchaeota)*	7.50	6.13	5.56	7.37	6.64	0.95
	*Crenarchaeota*	*Thermoprotei*	100.00	100.00	100.00	100.00	100.00	0.00
	*Thaumarchaeota*	*unclassified (derived from Thaumarchaeota)*	100.00	100.00	100.00	100.00	100.00	0.00
*Eukaryota*	*Streptophyta*	*Anthocerotopsida*	0.08	0.04	0.05	0.03	0.05	0.02
		*Bryopsida*	11.35	11.87	11.93	5.76	10.23	2.99
		*Charophyceae*	0.02	0.30	0.50	0.40	0.31	0.21
		*Chlorokybophyceae*	0.19	0.56	0.35	0.31	0.35	0.15
		*Coleochaetophyceae*	0.19	0.55	0.46	0.58	0.44	0.18
		*Coniferopsida*	0.10	0.17	0.13	0.07	0.12	0.04
		*Cycadopsida*	0.00	0.01	0.00	0.02	0.01	0.01
		*Equisetopsida*	0.00	0.00	0.00	0.01	0.00	0.00
		*Gnetopsida*	0.10	0.00	0.02	0.03	0.04	0.04
		*Isoetopsida*	5.66	6.06	5.70	2.59	5.00	1.62
		*Jungermanniopsida*	0.02	0.10	0.11	0.07	0.07	0.04
		*Liliopsida*	13.19	14.72	12.97	17.61	14.62	2.14
		*Lycopodiopsida*	0.02	0.00	0.02	0.00	0.01	0.01
		*Marattiopsida*	0.04	0.06	0.05	0.04	0.05	0.01
		*Marchantiopsida*	0.11	0.49	0.58	0.52	0.43	0.21
		*Mesostigmatophyceae*	0.31	0.30	0.57	0.29	0.37	0.13
		*Polypodiopsida*	0.15	0.13	0.06	0.03	0.09	0.06
		*Zygnemophyceae*	0.36	0.33	0.14	0.22	0.27	0.10
		*unclassified*	68.13	64.31	66.38	71.41	67.55	3.01
*Eukaryota*	*Ascomycota*	*Dothideomycetes*	3.76	4.72	3.97	9.25	5.42	2.58
		*Eurotiomycetes*	40.02	33.70	34.96	38.91	36.90	3.05
		*Leotiomycetes*	4.20	4.04	3.91	3.59	3.94	0.26
		*Pezizomycetes*	1.24	1.50	1.23	1.12	1.27	0.16
		*Pneumocystidomycetes*	0.02	0.01	0.01	0.00	0.01	0.01
		*Saccharomycetes*	22.78	26.30	25.60	19.83	23.63	2.95
		*Schizosaccharomycetes*	6.68	7.32	7.30	6.34	6.91	0.48
		*Sordariomycetes*	21.29	22.42	23.03	20.95	21.92	0.97
	*Chordata*	*Actinopterygii*	8.95	10.53	10.60	8.04	9.53	1.26
		*Amphibia*	20.18	11.76	11.01	36.08	19.76	11.65
		*Appendicularia*	0.02	0.02	0.00	0.03	0.02	0.01
		*Ascidiacea*	4.02	4.82	4.77	3.25	4.21	0.74
		*Aves*	5.92	8.13	6.94	4.24	6.31	1.65
		*Chondrichthyes*	0.00	0.00	0.02	0.00	0.00	0.01
		*Mammalia*	51.71	54.93	56.16	41.87	51.17	6.48
		*Thaliacea*	0.00	0.00	0.02	0.00	0.00	0.01
		*unclassified (derived from Chordata)*	9.19	9.81	10.48	6.50	9.00	1.75
	*Arthropoda*	*Arachnida*	9.97	8.11	7.89	8.18	8.54	0.96
		*Branchiopoda*	0.00	0.00	0.03	0.05	0.02	0.03
		*Cephalocarida*	0.00	0.00	0.00	0.00	0.00	0.00
		*Diplopoda*	0.00	0.00	0.00	0.00	0.00	0.00
		*Diplura*	0.00	0.00	0.00	0.00	0.00	0.00
		*Ellipura*	0.00	0.00	0.03	0.00	0.01	0.02
		*Insecta*	90.03	91.77	92.04	91.63	91.37	0.91
		*Malacostraca*	0.00	0.09	0.00	0.11	0.05	0.06
		*Maxillopoda*	0.00	0.03	0.00	0.03	0.01	0.02
		*Merostomata*	0.00	0.00	0.00	0.00	0.00	0.00
		*Ostracoda*	0.00	0.00	0.00	0.00	0.00	0.00
	*Bacillariophyta*	*Bacillariophyceae*	44.06	53.78	53.02	44.07	48.73	5.40
		*Coscinodiscophyceae*	55.94	45.85	46.24	54.81	50.71	5.41
		*Fragilariophyceae*	0.00	0.36	0.73	1.13	0.56	0.48

**Figure 2:**
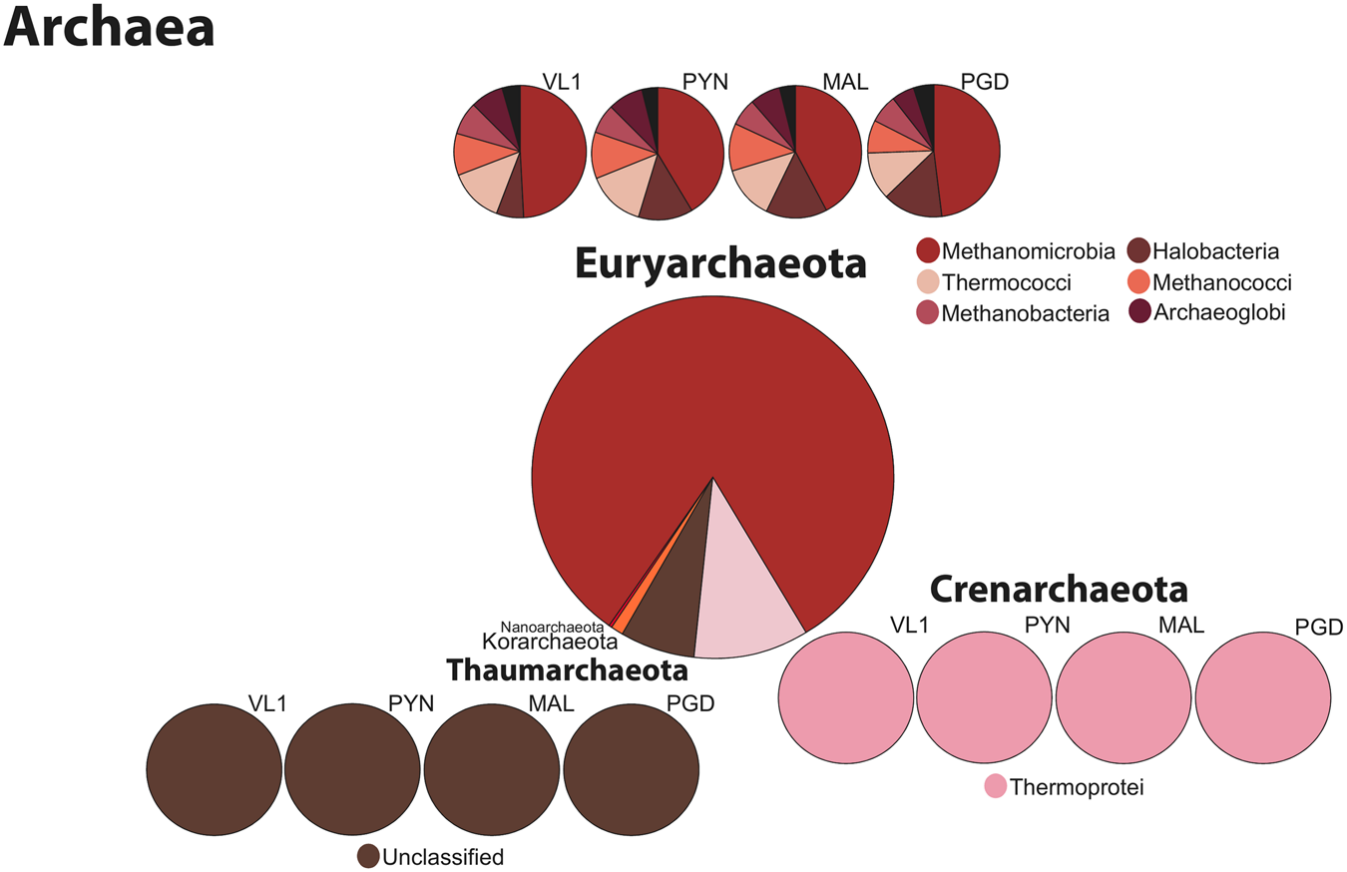
Taxonomic structure and diversity of the reads mapping to *Archaea*. Kingdom, phyla and class divisions across the four sampling locations in Kerala, India, with class divisions noted across the top three phyla.

**Figure 3:**
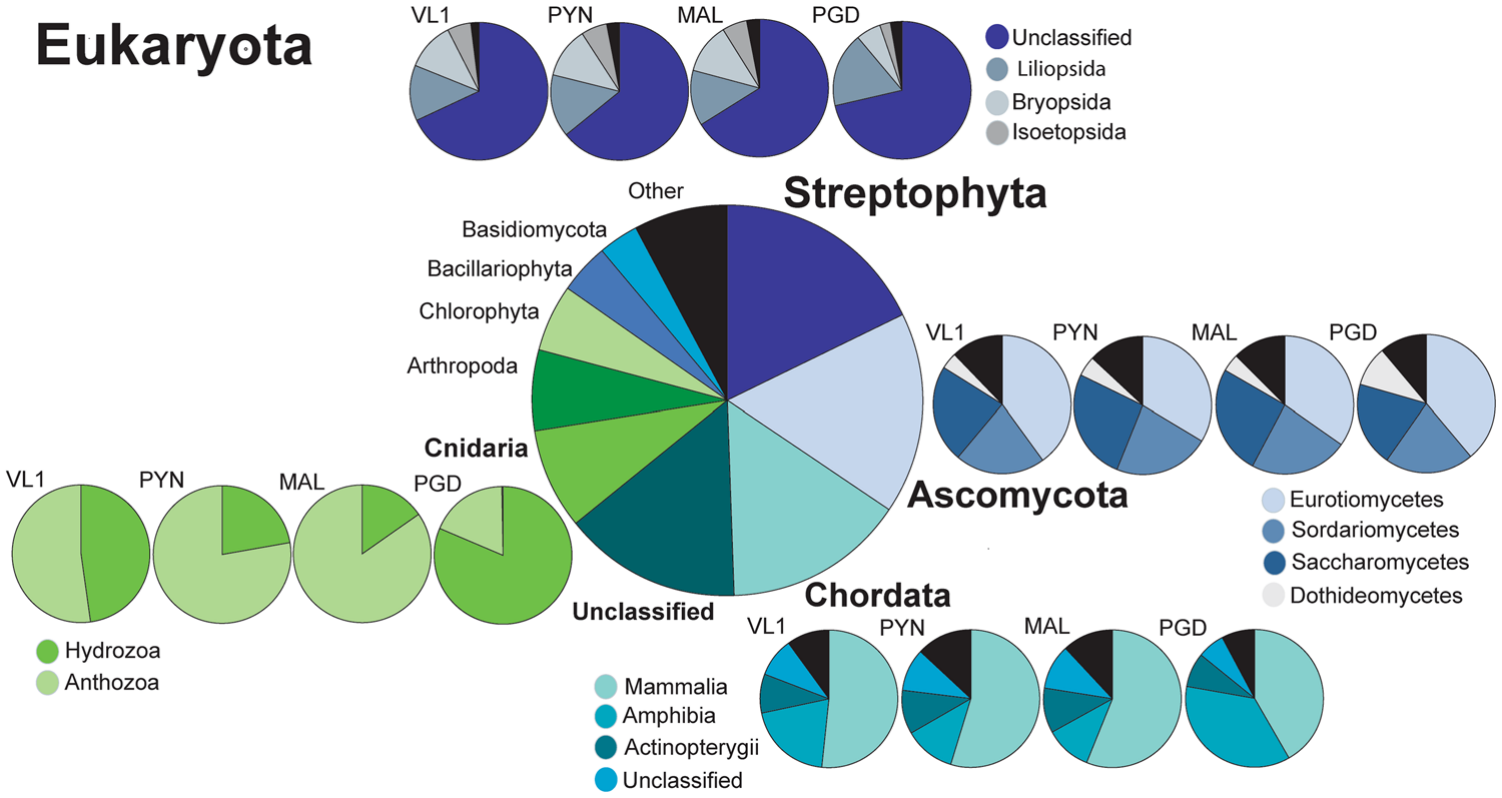
Taxonomic structure and diversity of the reads mapping to *Eukaryota*. Kingdom, phyla and class divisions across the four sampling locations in Kerala, India, with class divisions noted across the top four phyla.

A majority of the reads assigned to *Archaea* (1.32 ± 0.44% of total reads) mapped to the phylum *Euryarchaeota* (81.77% ± 7.02) across all four samples (Fig. 2 and Table 2). The remainder of the reads in *Archaea* mapped predominantly to *Crenarchaeota* (10.43% ± 2.16) and *Thaumarchaeota* (6.43 ± 8.47), although the order and the percentage varied across the four Kerala locations. Of the 1.32% of the assigned reads that mapped to *Eukaryota*, 90% of those reads were found across 8 phyla (Fig. 3 and Table 2), with the majority (18.04% ± 3.55) mapping to the phylum *Streptophyta*, closely followed by *Ascomycota* (16.4% ± 1.78) in all of the samples except PGD. Unlike what was seen in *Bacteria* and A*rchaea*, a large number of the *Eukaryota* reads (14.94% ± 0.88) could not be classified to a specific phylum.

**Figure 4:**
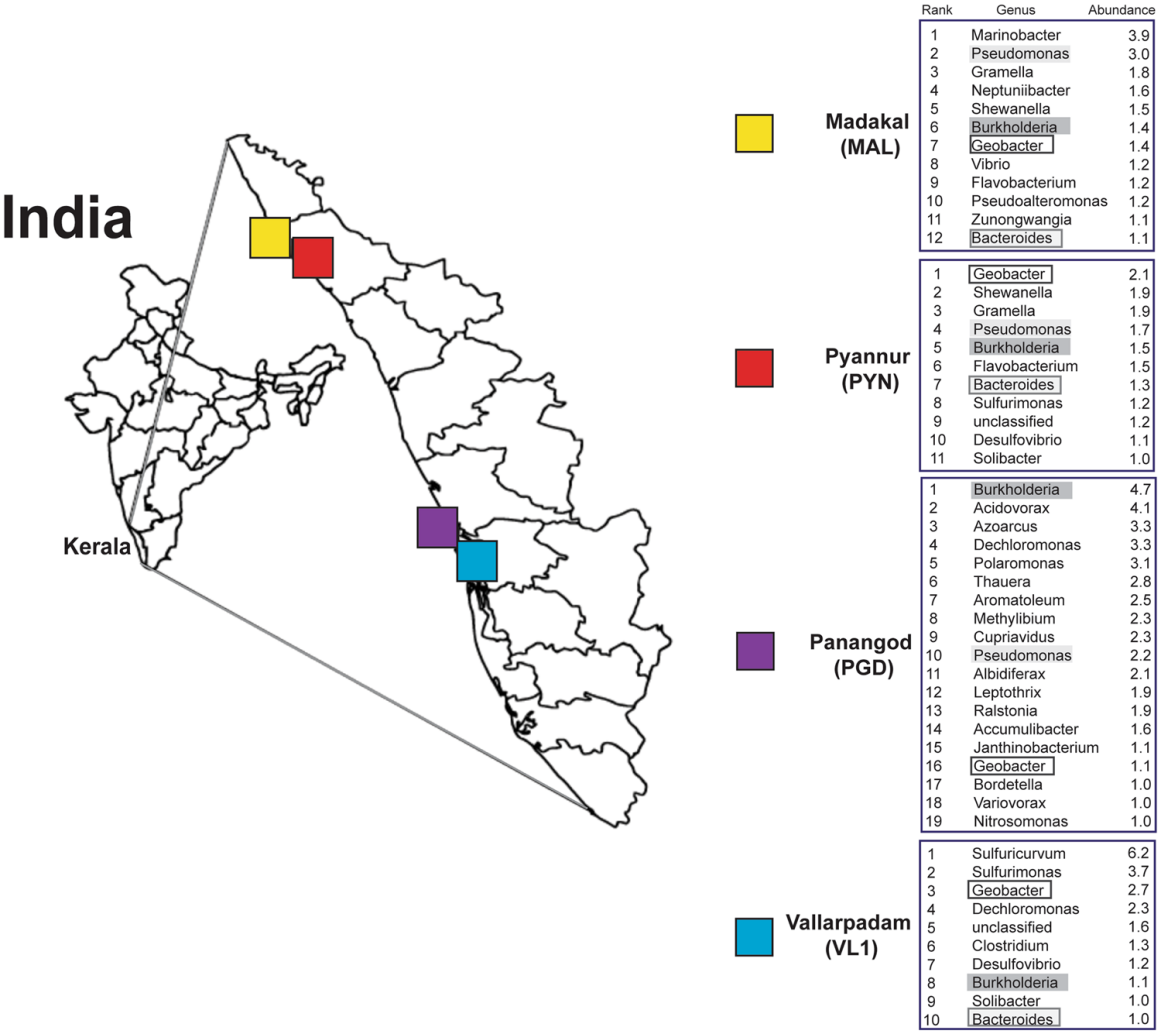
Geographic locations and diversity in the sampling sites in the mangrove ecosystem of Kerala, India. Genera with more than 1% of the mapped reads are listed, with their rank and indications of their abundance are shown, and organisms shared across sampling locations are indicated by shading or boxes. (Image source: http://www.d-maps.com/carte.php?num_car=24853&lang=en andInkscapev0.92 was used for sample site labelling).

An analysis of the genera found in these sediment samples showed unique differences at each sampling location, with some similarities. The reads that mapped to each genus were calculated, and only those genera with more than 1% of the total mapped reads were noted (Fig. 4). *Burkholderia* and *Geobacter* were the only genera that were found in each of the sampling locations. Each location had a different predominant genus, and while three locations had most of the same dominant genera (PYN, MAL and VL1), PGD was quite different in both the number and members identified.VL1 had *Sulfuricurvum*, which at 6% had almost double the reads as the next genus (*Sulfurimonas*). *Burkholderia* was the most prominent in PGD, with *Acidovorax* also prominent. *Geobacter, Gramella* and *Shewanella* were the dominant genera found at the PYN location, and *Marinobacter* was the most prominent in MAL.

### Comparison of metagenomes from different soil samples

**Figure 5:**
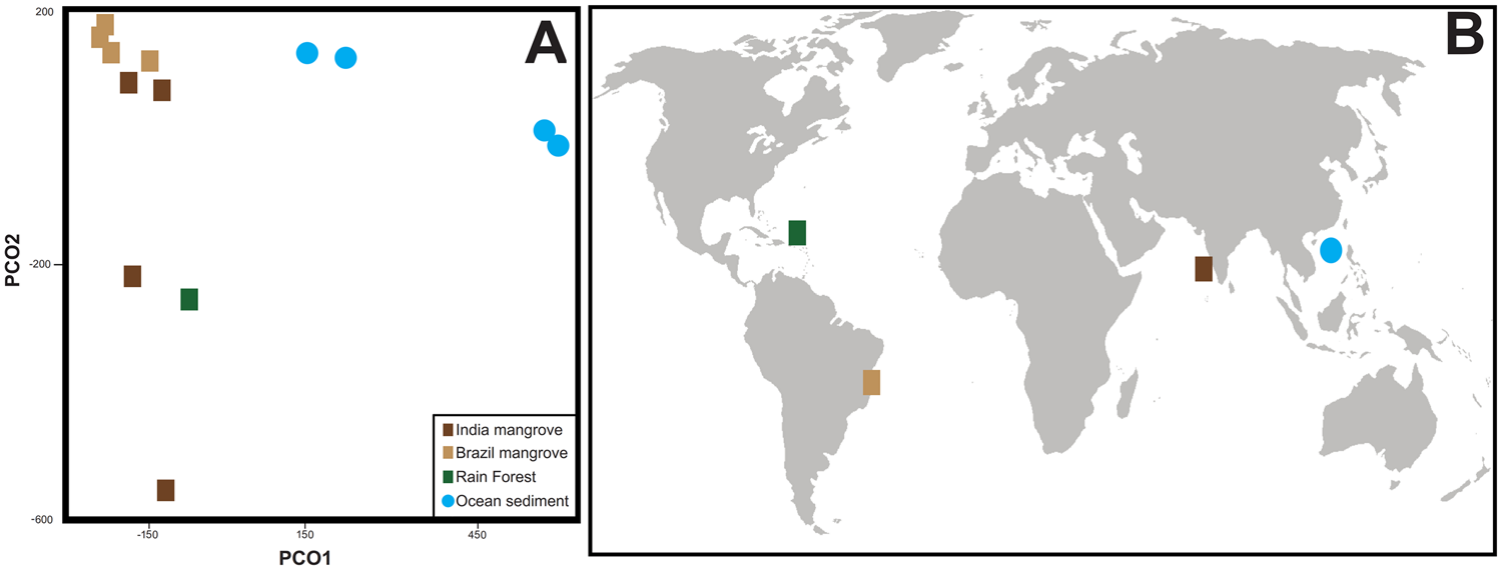
Principal Component Analysis and geographic location of samples compared across the globe. A. PCA analysis comparing mangrove forests, a rain forest, and four ocean sediment samples. B. Geographic locations of the samples that were compared in this analysis. (Image source- Link:https://commons.wikimedia.org/wiki/File:BlankMap-World-noborders.pngand Inkscape v0.92 was used for labelling).

Sediment samples from three different ecosystems were compared to the Kerala mangrove samples. These included four samples from a Brazilian mangrove forest^21^, sediment from a tropical rain forest in Puerto Rico^22^, and four ocean sediment samples from the South China Sea^23^. A Principal Component Analysis (PCA) of all the samples clearly grouped the forest isolates together on one axis, clearly separating them from the ocean samples (Fig. 5).

**Figure 6:**
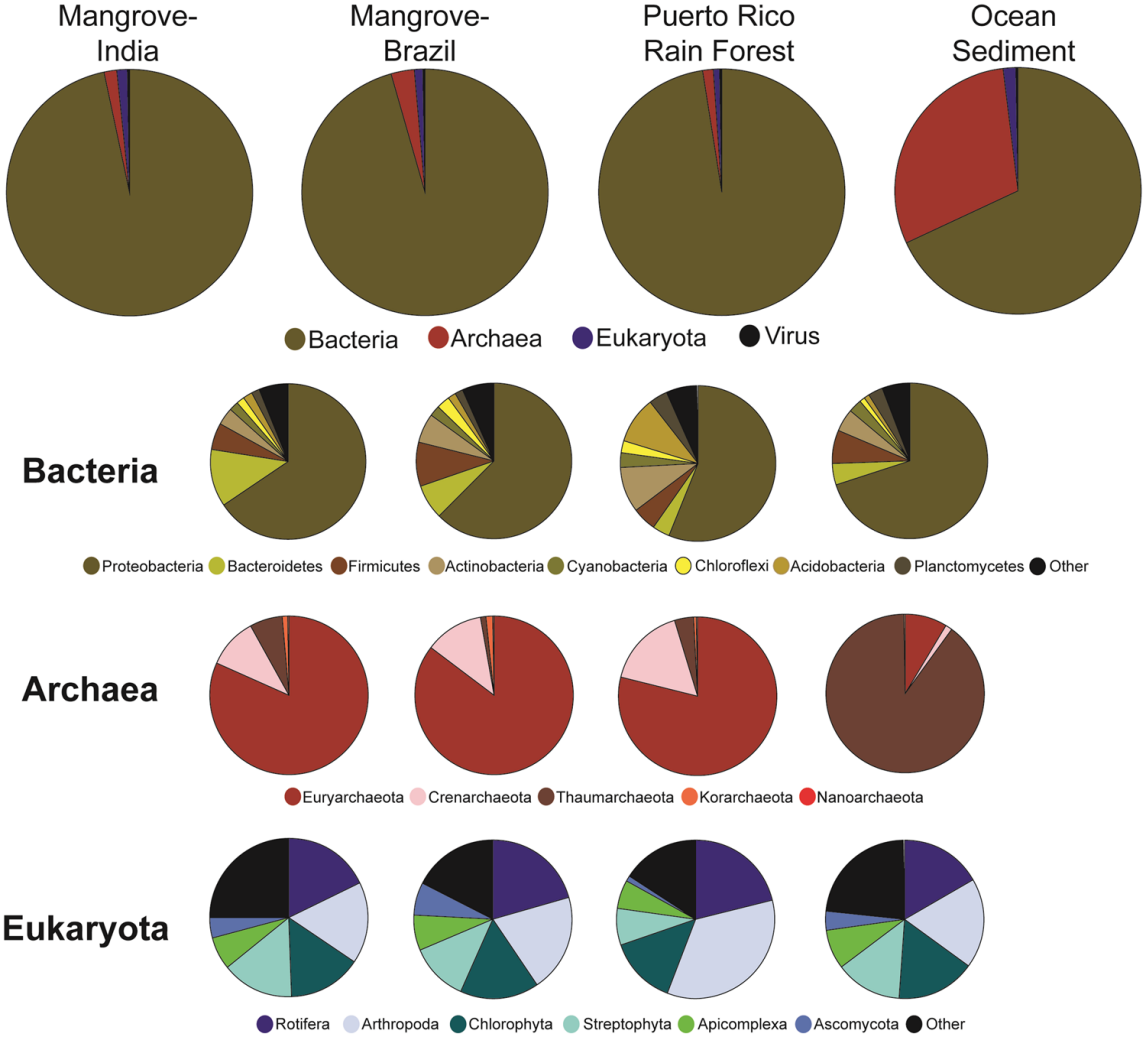
Taxonomic structure at the highest taxonomic levels across all samples. Averages of reads across samples from the same geographic location are pictured, with diversity at the kingdom level (large circles), different phyla within those kingdoms (smaller circles), and classes presented.

MG-RAST’s organism tree tool was used to examine patterns across the different sediment types. At the highest taxonomic level (Super Kingdom) clear differences were seen between the ocean sediment samples, and those isolated from the two types of forests. The four ocean samples had an average of 868769.5 (±123583) reads, with an average of more than 99% assigned to a taxonomic category (Table 1 and Fig. 6). In contrast, the majority of the reads isolated from any of the forest samples mapped to *Bacteria*, with *Archaea* being less than 5% of the assigned total in any sample (Table 1 and Fig. 6). All of the sediment samples had approximately 1.5% of the reads assigned to *Eukaryota*. Very few virus reads were found.

A more detailed look at the two taxonomic levels with the majority of the reads assigned (*Archaea* and *Bacteria*) showed some striking differences between the assigned phyla within these two groups. Most notably, the ocean sediment samples had the highest archaeal reads, while all of the arboreal samples are predominantly bacterial (Table 1). An average 9. 3% of the ocean reads mapped to *Archaea*, and more than 95% of the arboreal reads, both mangrove and rain forest, are bacterial. Those bacterial reads, regardless of the ecology of the samples, map to *Proteobacteria* across all samples (Fig. 6), but that similarity does not extend beyond the phylum. Within this phylum, the Kerala mangrove samples have either *Gamma*- or *Betaproteobacteria* as the class that has the most reads assigned to it, with the Brazilian mangrove and Puerto Rican forest samples both having *Gammaproteobacteria* as the dominant class (Supplementary Table 4). The ocean samples have either *Beta*- or *Alphaproteobacteria* as the most dominant class. The lack of consensus, both within and between the groups, is also seen with classes within the *Bacteriodes* and *Firmicutes* phyla (Supplementary Table 4). The only bacterial phylum where all groups shared a similar structure was the *Actinobacteria*, with *Actinomycetales* as the dominant class (Supplementary Table 4).

The ocean sediments had a high abundance of reads that map to the archaeal kingdom and within that, *Thaumarchaeota* as the dominant phylum (Fig. 6). *Archaea* reads were less than 5% of the total from the samples isolated from forests, but across all of them, *Euryarchaeota* was the dominant phylum, followed by *Crenarchaeota*. All of the samples, both from the mangrove and rain forests, had *Methanomicrobia* as the dominant class in *Euryarchaeota*. They also shared *Thermoprotei* as the most prevalent *Crenarchaeota* class. Reads that mapped to these particular classes were not even found (or barely registered) in any of the ocean samples, which instead had *Thermococci* and *Methanococci* as the most prevalent classes among the ocean reads that mapped to the *Euryarchaeota* phyla.

Less than 2% of the total reads from any sample mapped to *Eukaryota*. Most of these reads mapped to *Streptophyta* in the Indian and Brazilian samples, but the majority of these could not be classified to any particular class (Supplementary Table 4). The dominant class in the sample from the rain forest was *Ascomycota. Streptophyta* was also an important part of the ocean eukaryotic reads, but the *Bacillariophyta*, which contains the diatoms, was the dominant phylum. There was no clear consensus in a dominant class across the groups.

### Functional analysis

A functional comparison that mapped the abundance profiles to different categories across the Subsystems^24^ was performed for each of the Indian mangroves samples. All four samples had similar distributions and abundance of reads that were mapped at the highest levels of subsystem categorization (Supplementary Table 2).

**Figure 7:**
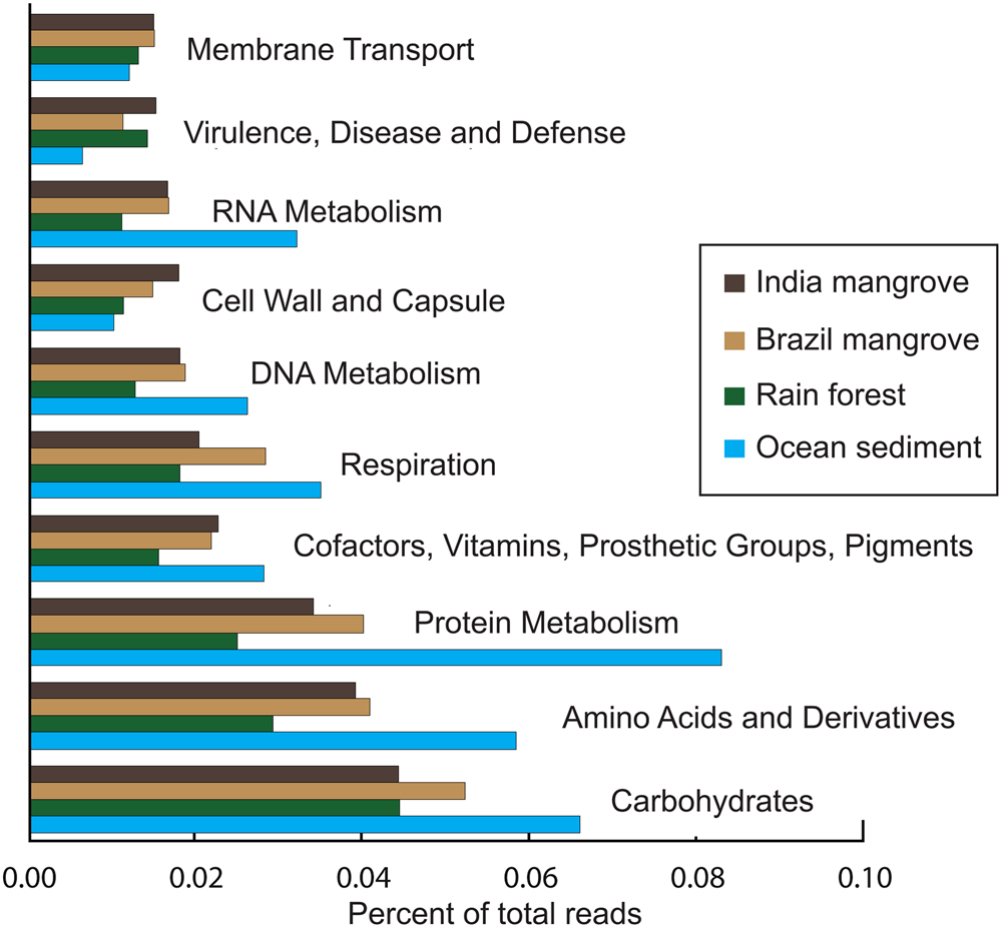
Subsystem functional analysis of representatives from each of the ecosystems analyzed showing the top ten subsystems that the reads were assigned to. Reads that map to genes assigned to specific subsystems are demonstrated for a single representative from each geographic location compared.

A single sample was chosen from each of the groups for a functional comparison (India mangrove = PGD/4671368.3, Brazil mangrove = BrMgv2/4451034.3, Puerto Rico Forest = 4446153.3, South China Sea = E201-2/4487295.3). The numbers of hits for each of the functional levels were summed, and the percentages of hits per each level compared to the total number were compared (Supplementary Table 3 and Fig. 7). The forest samples had similar profiles, the exception being Protein Metabolism, where the India mangrove sample (PGD) had many fewer reads (Supplementary Table 2), and the ocean sample with a clear majority of mapping reads. The subsystems that had the highest Z score when comparing the average across the three forest samples to the ocean sample were Carbohydrates, Clustering-based subsystems, Protein metabolism and Amino acids derivatives (Supplementary Table 3 and Fig. 7).

## Discussion

### Similarities and differences seen across the Kerala mangrove samples

All of the Kerala mangrove samples shared certain microbial community structures. *Bacteria* were the predominant organisms, averaging 81.72% of the total reads across all four locations. Metagenomic analysis of mangrove at Cardoso Island State Park, Brazil, through 16S rRNA pyrosequencing showed similar dominance of *Proteobacteria* (88% of overall sequence) irrespective of soil depth through 16S rRNA pyrosequence^25^. Within the *Bacteria*, *Proteobacteria* was the dominant phylum, followed by *Bacteriodes*, *Firmicutes* and then *Actinobacteria*. Similar bacterial phylum dominancy was found using PCR-Clone based metagenomic library screening^26^ as well as 16S rRNA ribo-typing^27^. The similarities within Kerala datasets seen across the higher taxonomic levels (Kingdom and Phylum) did not continue at the class level, where the composition of the bacteria varied at each location (Fig. 1), especially among the classes within the *Proteobacteria* and *Bacteriodes* phyla. Archaea followed next with the most reads, but with an average of 1.8%, they were not considered a dominant member of the Kerala microbial community. Despite their small numbers, they had a remarkable consistency in their taxonomic structure, maintaining the same divisions even to the class level (Fig. 2). Previous whole metagenome study in Brazilian mangroves sediments by Andreote *et al*.^21^ have found similar abundance (0–3.4%) of archaea. Furthermore, mangrove soil sediment from Saudi Arabia^28^ has also showed similar percentage abundance (3.5%). The consistent level of archaea in mangrove samples could denote its importance in the ecosystem such as N cycle by *Thaumarchaeota* and the favorable conditions for Methanogens^29^. *Eukaryota* had almost as many reads assigned to it as the *Archaea*, with 1.32% of the total, and all four samples had similar distributions at the taxon class level (Fig. 3).

Genera that had more than 1% of the reads mapped to them were examined at each sampling location, revealing some similarities and differences across the Kerala isolates (Fig. 4). Only two genera, *Burkholderia* and *Geobacter*, had more than one percent of the sequences map to them in each of the four samples. The mangrove samples from Kerala (India) were affected by anthropogenic activities, hence, it is very likely that the high dominance of *Burkholderia* could be due to its role involved in degradation of various compounds such as polycyclic aromatic hydrocarbons (PAHs), diesel, kerosene, naphthalene, and phenol^30,31^. *Burkholderia sp*. was also abundant in Okinawa (Japan) oil contaminated mangrove sediments^32^. Three of the locations (MAL, PYN and VL1) shared most of the genera that had more than 1% of the total reads, but those found in PGD at similar levels were mostly unique. The two predominant genera found in the VL1 were *Sulfuricurvum* and *Sulfurimonas*, with 6.2% and 3.66% of the total reads mapping to them. Both of these genera have been associated with sulfur oxidation^33–37^. *Sulfuricurvum* is in high concentration only in VL1, but *Sulfurimonas* is also a predominant organism in PYN, with 1.19% of the reads mapping to this genus in this sample. MAL and PYN were in close proximity compared to the other samples, and they shared *Marinobacter* as a dominant genus (with 3.93% and 0.79% of the reads, respectively). *Marinobacter* has been described as being ubiquitous across the global oceans^38^, and is known to degrade hydrocarbons^39^ and fix nitrogen^40^. This genus is also of interest as it has been shown to be one of the few bacterial genera known to “bloom” when oil or oil constituents are introduced into seawater^41^, and its predominant presence in the co-located MAL and PYN, as compared to VL1 and PGD could suggest some pollution in that environment when the samples were taken.

Although there were differences in the bacterial membership at certain taxonomic levels, this was not repeated when the functional capacity of the four samples was compared. The reads across all four samples had similar abundance patterns that were assigned to the 28 different functional categories used by Subsystems^22^. No significant differences were seen in sulfur metabolism, despite VL1 having the two most abundant genera being sulfur oxidizers.

### Similarities and differences across global samples

To look for similarities that might be shared across similar ecosystems, the Indian mangrove samples were compared with four samples isolated from mangrove forests in Brazil^21^, a tropical forest in Puerto Rico^22^ and four samples collected sediment from the South China sea^23^. A principal component analysis clustered the samples taken from forests, which includes both the mangrove samples and the tropical rain forest isolate, closer to the y-axis than those samples collected in the ocean (Fig. 5). A taxonomic analysis revealed supported this finding. At the highest taxonomic levels, all of the forest samples had similar taxonomic patterns (Fig. 6), with the ocean samples being distinctly different. *Bacteria* were the dominant kingdom in all samples taken from forests with Archaea less than 5%, however, the ocean isolates have almost 12.9% archaeal reads. Previously, more than 87% of the microbial biomass was seen to be dominated by Archaea in deep subsurface sediments^42^. In addition, Antarctic circumpolar continental shelf waters have been shown to be highly dominated by Archaea^43^. Dominance of archaea in deep ocean subsurface and bacteria in arboreal samples could be explain according to the theory proposed by Valentine^44^ which states that bacteria can adapt to the changing environment while archaea can sustain in the nutrient limited environment. At the phylum taxon, all forest samples were predominantly *Proteobacteria*. This was also true of the ocean samples, but as they accounted for less than 10% of the total reads they were not the dominant ocean phylum. Nevertheless, *Proteobacteria* was found to be the most dominant in all the ecosystems within the bacterial kingdom. Saline environments have shown to harbor *Proteobacteria* in the past^45,46^. The mangrove samples from Brazil and India had a mixture of *Bacteriodes*, *Firmicutes* and *Actinobacteria* as a significant part of the bacterial phyla diversity, which corroborates to mangroves data generated from other part of India^47^, but the Puerto Rican rainforest had two additional phyla (*Acidobacteria* and *Planctomycetes*) that were only a small part of the diversity in the mangroves. *Acidobacteria* have been found to be dominant in rain forest sample^48,49^. Ocean samples, which also included reads that mapped to *Bacteriodes*, *Firmicutes* and *Actinobacteria*.

Similarities were also seen in the structure of the *Archaea* across the four ecosystems examined. Of the *Archaea* reads that were present, the forest samples contained mainly *Euryarchaeota*. The ocean samples were primarily archaeal and mostly of the phylum *Thaumarchaeota*. The finding is in corroboration with the work of Quaiser *et al*.^50^, which showed the dominance of archaea by Group-I *Thaumarchaeota* in the sediment of Marmara Sea. They found that the archaeal *amo* (ammonia mono-oxygenase) genes were highly abundant in Marmara Sea suggestion the dominance of *Thaumarchaeota* in ammonia oxidation. Marine Group-I (MG) group were also found to be the most dominated group among the archaeal kingdom in deep Mediterranean Sea^51^. Studies have shown that the seasonal variation affects the archaeal diversity wherein Marine Group-I (MG) and *Euryarchaeota* MG II.b dominates during winter and *Euryarchaeota* MG II.b during summer^52^. Archaeal diversity can also be influenced by the difference in zones (depth) of the sea/ocean; the oxic/anoxic interface zone featured high dominance of Marine Group-I (MG) archaea. However, significant reduction was exhibited in sulfatemethane transition zone and methanic zone^53^. Similar patterns were also seen in the *Eukaryota*, which were not in the majority in any sample. Across both the forest and ocean samples the *Streptophyta*, which include the green plants, were a predominant phylum found in the eukaryotic reads. The forest samples also had reads that mapped to the phyla *Ascomycota* (fungi) in significant numbers and a large number in *Chordata* (vertebrates), *Cnidaria*, and *Arthropoda* (insects and arachnids). These same phyla were also present in the ocean. The mangrove and ocean samples had a number of reads mapping to the phylum *Bacillariophyta* (data not shown), which include the diatoms. *Bacillariophyta* play a crucial role in generation of organic carbon soluble in organic compounds in the ocean bottom and also produces exopolysaccharides (EPS) which stabilizes the sedimentary materials^54^.

A similar pattern that groups the forest samples distinctly from the ocean isolates was also seen in the functional analysis. While all samples had similar functional profiles in some of the subsystems, the forest samples were clearly distinct from the ocean samples in several of the subsystems examined. Interestingly, the ocean samples examined had more reads that mapped to genes active in the subsystems defined as Carbohydrates, Amino Acids and Derivatives, Protein Metabolism, Respiration, and RNA Metabolism (Fig. 7), but had fewer reads that mapped to the Virulence, Disease and Defense.

## Conclusions

A metagenomic analysis of isolates from soil sediment in four different locations across the Kerala, India mangrove forest ecosystem showed strong similarities across higher taxonomic divisions extending to the level of phylum. Comparisons of the Indian mangrove isolates to samples from mangrove in Brazil, and to a tropical rain forest in North America showed similar patterns, with most of the reads mapping to phylum *Proteobacteria* within the *Bacteria* kingdom. Fewer reads were found that mapped to *Archaea*, but those that were present predominantly to the phylum *Euryarchaeota*. Similar numbers of reads in each of the isolates from mangrove or rainforest, mapped to *Eukaryota*, which showed comparable divisions across the phyla S*treptophyta* (green plants), *Ascomycota* (fungi), *Chordata* (vertebrates), *Cnidaria* and *Arthropoda* (insects and arachnids). However, differences were noted, when these samples were compared to isolates taken from ocean sediments. The ocean samples had, on average, larger number of *Archaea*, with almost all the reads mapping to the phylum *Thaumarchaeota*. Like the forest samples, the predominant bacterial phylum in the ocean samples mapped to the phylum *Proteobacteria*.

This finding shows strong patterns in metagenomics structure across the samples taken from the two types of forest ecosystems. It shows distinct patterns that unite the forest samples and differentiates them from an ocean sediment ecosystem.

## Methods

### Soil samples

Approximately 250 g of soil were collected at 20 cm in depth from the surface in four different locations within Kerala, India and included Pyannur (PYN, Coordinates: 12.1050687, 75.2058), Panangod (PGD, Cord: 9.8959941, 76.326094), Vallarpadam(VL1, Cord: 9.9994138, 76.253705) and Madakal (MAL, Cord: 9.9091896, 76.30629) (Fig. 4B). Physical characteristics of the samples sites are provided (Supplementary Table 4). Following collection, each soil sample was preserved at −20 °C prior to DNA isolation.

### Genomic DNA extraction and Sequencing

Genomic DNA was extracted using MoBio Powersoil DNA isolation Kit (MO BIO Laboratories, Inc., California) as per manufacturer’s instructions. Sample quality was verified by gel electrophoresis. Paired end read sequencing was performed on an Illumina HiSeq platform 2500 at SciGenome Labs Pvt Ltd, Cochin (India). The raw fastQ files were uploaded in NCBI SRA database with accession numbers SRR2844600, SRR2844601, SRR2844602 and SRR2844616.

### Quality Control and Annotation Pipeline

The paired end fastQ read files of all the samples were uploaded to the Metagenome Rapid Annotation using Subsystem Technology (MG-RAST) server (http://metagenomics.anl.gov/)^55^ and processed following their standard protocol. Briefly, the mate-pairs were joined with overlap setting of 8 base pairs (bp) and a maximum difference of 10% and were then processed for quality control. Low-quality regions were trimmed off using SolexaQA^56^, de-replicated, and analyzed the artificially duplicated reads (ADRs)^57^ using Duplicate Read Inferred Sequencing Error Estimation (DRISEE)^58^. The near-exact matches against model organisms including fly, mouse, cow and human were removed using Bowtie^59^. Coding regions in DNA sequences of 75 bp and longer were predicted using FragGeneScan^60^. Protein clusters with 90% identity were built using the UCLUSTimplementation^61^ in Quantitative Insights into Microbial Ecology (QIIME). A representative of each cluster is subjected to similarity analysis using BLAST-like alignment tool (BLAT)^62^. Sequence similarity searches to identify proteins and mapped annotations are computed against a MG-RAST protein databases M5NR^19^, Genbank^63,64^, SEED^23^, Integrated Microbial Genomes & Microbiomes (IMG)^65^, Universal Protein Resource (UniProt)^40^, Kyoto Encyclopedia of Genes and Genomes (KEGG)^66^ and Evolutionary genealogy of genes: Non-supervised Orthologous Groups (eggNOG)^67^.

### Taxonomic and Functional Analysis

Taxonomic assignments were carried out against the RefSeq protein database, Metagenomics Rapid Annotation using Subsystem Technology (MG-RAST)^55^. This database is an integration of many sequence databases into one single, searchable database. Cut-offs included a maximum E-value of 1 × 10^−5^, a minimum percentage identity of 60%, and a minimum alignment length of 15 were used. The subsystems platform annotation that assigns genes to functional roles^68,69^, was used for functional analysis comparisons, with the same cut-off values used for the taxonomic assignments.

### Comparison of metagenomes from different soil samples

Different sediment samples isolated from mangroves in Brazil^21^, a tropical forest in Puerto Rico^22^, and different sediment samples collected from the South China Sea^23^ were compared to the Kerala, India soil samples (Table 1), which were publically available at MG-RAST. We selected samples that could be linked to public data. A PCA provided by MG-RAST was used to examine dimensions of maximal variation, and an examination of the taxonomic diversity across all samples was conducted using the Organism tree tool. Taxonomic assignments used M5NR with the same cut-offs used for the Kerala samples, and an average was made across all the samples from a similar location/ecosystem for final comparison. The subsystems platform was used to assigned functional roles, with settings similar to those described above. When functional comparisons were made across metagenomes from geographic and ecological variants, a single isolate that was chosen based on the number of reads to represent each of the geographic groups (India-PGD-4671368.3, Brazil-BrMgv2-4451034.3, Puerto Rico Rain forest- 4446153.3, South China Sea-E201-1-4487294.3).

### Availability of data and materials

The raw fastQ files for the Kerala India samples were uploaded in NCBI SRA database with accession numbers SRR2844600, SRR2844601, SRR2844602 and SRR2844616. In addition, the sequences for these reads are publicly available from the MG-RAST server under the following IDs: 4671371, 4671370, 467136 and 4671368.

## Electronic supplementary material

Supplementary Dataset 1
