# Author Correction: Searching for signatures across microbial communities: Metagenomic analysis of soil samples from mangrove and other ecosystems

**DOI:** 10.1038/s41598-017-18550-0

**Published:** 2018-01-04

**Authors:** Madangchanok Imchen, Ranjith Kumavath, Debmalya Barh, Vasco Azevedo, Preetam Ghosh, Marcus Viana, Alice R. Wattam

**Affiliations:** 1 Department of Genomic Science, School of Biological Sciences, Central University of Kerala, Periye, Padanakkad P.O., Kasaragod, Kerala 671314 India; 2 Centre for Genomics and Applied Gene Technology, Institute of Integrative Omics and Applied Biotechnology, Nonakuri, Purba Medinipur, West Bengal 721172 India; 3 Xcode Life Sciences, 3D Eldorado, 112 Nungambakkam High Road, Nungambakkam, Chennai, Tamil Nadu 600034 India; 4 Laboratório de Genética Celular e Molecular, Departamento de Biologia Geral, Instituto de Ciências Biológicas (ICB), Universidade Federal de Minas Gerais, Pampulha, Belo Horizonte, Minas Gerais Brazil; 5 Department of Computer Science, Virginia Commonwealth University, Richmond, Virginia 23284 USA; 6 Biocomplexity Institute, Virginia Tech University, Blacksburg, Virginia 24061 USA

Correction to: *Scientific Reports* 10.1038/s41598-017-09254-6, published online 18 August 2017

The original version of this Article contained a typographical error in the spelling of the author Azevedo V, which was incorrectly given as Avezedo V. This has now been corrected in the PDF and HTML versions of the Article, and the Supplementary Information file that accompanies the Article.

