# Supplementary material for: Searching for signatures across microbial communities: Metagenomic analysis of soil samples from mangrove and other ecosystems
Source: Sci Rep. 2017 Aug 18;7:8859. doi: 10.1038/s41598-017-09254-6 (PMC5562921; doi:10.1038/s41598-017-09254-6)
Supplement: Supplementary file 1 — Supplementary Dataset 1 [file 41598_2017_9254_MOESM1_ESM.pdf]

Searching for signatures across microbial communities: Metagenomic analysis of soil samples from mangrove and other ecosystems

Madangchanok Imchen[1], Ranjith Kumavath[1*], Debmalya Barh[2, 3,4], Vasco Azevedo[3], Preetam Ghosh[4], Marcus Viana[3], Alice R. Wattam[5*]

Supplementary Table 1.The number of hits and the percentage of the total assigned to different phyla in bacteria, archaea and eukaryote across the mangrove soil samples isolated from four different locations in Kerala, India.

Kingdom	Phylum	VL1		PYN		MAL		PGD		Total	Average	Average Percentage
		Hits	%	Hits	%	Hits	%	Hits	%			
Bacteria	*Proteobacteria*	1974270	64.14	1896950	57.33	1960225	62.50	2609790	78.51	8441235	1688247	65.71
	Bacteroidetes	192693	6.26	566086	17.11	512942	16.36	248748	7.48	1520469	304093.8	11.84
	Firmicutes	272098	8.84	205922	6.22	156109	4.98	80246	2.41	714375	142875	5.56
	Actinobacteria	90595	2.94	126676	3.83	112923	3.60	133685	4.02	463879	92775.8	3.61
	Cyanobacteria	74010	2.40	74728	2.26	64831	2.07	38123	1.15	251692	50338.4	1.96
	Chloroflexi	97696	3.17	68480	2.07	47873	1.53	26234	0.79	240283	48056.6	1.87
	Verrucomicrobia	66682	2.17	74755	2.26	54286	1.73	42280	1.27	238003	47600.6	1.85
	Acidobacteria	66738	2.17	59858	1.81	49146	1.57	57521	1.73	233263	46652.6	1.82
	Planctomycetes	53727	1.75	71955	2.17	56095	1.79	19016	0.57	200793	40158.6	1.56
	Chlorobi	37190	1.21	37958	1.15	30769	0.98	11894	0.36	117811	23562.2	0.92
	Spirochaetes	20043	0.65	17493	0.53	12277	0.39	17394	0.52	67207	13441.4	0.52
	Deinococcus-Thermus	18040	0.59	18461	0.56	14982	0.48	9517	0.29	61000	12200	0.47
	Nitrospirae	16409	0.53	12936	0.39	8098	0.26	5301	0.16	42744	8548.8	0.33
	Thermotogae	17019	0.55	12226	0.37	8422	0.27	3288	0.10	40955	8191	0.32
	Aquificae	15930	0.52	9602	0.29	7239	0.23	2747	0.08	35518	7103.6	0.28
	Deferribacteres	9260	0.30	7597	0.23	4862	0.16	1497	0.05	23216	4643.2	0.18
	Synergistetes	9956	0.32	6614	0.20	4572	0.15	1644	0.05	22786	4557.2	0.18
	Gemmatimonadetes	5763	0.19	7569	0.23	5614	0.18	3660	0.11	22606	4521.2	0.18
	unclassified	8156	0.26	6088	0.18	4805	0.15	3019	0.09	22068	4413.6	0.17
	Fusobacteria	6635	0.22	5904	0.18	4932	0.16	1876	0.06	19347	3869.4	0.15
	Lentisphaerae	6042	0.20	5357	0.16	3218	0.10	1151	0.03	15768	3153.6	0.12
	Chlamydiae	4456	0.14	4360	0.13	3834	0.12	1688	0.05	14338	2867.6	0.11
	Dictyoglomi	5282	0.17	3121	0.09	2225	0.07	827	0.02	11455	2291	0.09
	Chrysiogenetes	3013	0.10	2416	0.07	1816	0.06	858	0.03	8103	1620.6	0.06
	Candidatus Poribacteria	1598	0.05	1930	0.06	1319	0.04	495	0.01	5342	1068.4	0.04
	Elusimicrobia	2201	0.07	1500	0.05	1002	0.03	448	0.01	5151	1030.2	0.04
	Fibrobacteres	1218	0.04	1165	0.04	817	0.03	614	0.02	3814	762.8	0.03
	Tenericutes	1132	0.04	1093	0.03	973	0.03	379	0.01	3577	715.4	0.03
Archaea	*Euryarchaeota*	100385	86.79	46898	78.69	32535	73.97	13333	78.74	193151	48287.75	81.78
	Crenarchaeota	11937	10.32	6345	10.65	4167	9.47	2198	12.98	24647	6161.75	10.44
	Thaumarchaeota	1627	1.41	5543	9.30	6821	15.51	1218	7.19	15209	3802.25	6.44
	Korarchaeota	1436	1.24	650	1.09	350	0.80	167	0.99	2603	650.75	1.10
	Nanoarchaeota	285	0.25	164	0.28	112	0.25	18	0.11	579	144.75	0.25
Eukaryota	*Streptophyta*	5233	18.49	6965	16.21	6338	16.12	13340	20.20	31876	7969	18.05
	Ascomycota	5237	18.50	7419	17.27	6757	17.18	9697	14.69	29110	7277.5	16.48
	Chordata	4579	16.18	6532	15.21	5705	14.51	9632	14.59	26448	6612	14.97
	unclassified	3909	13.81	6084	14.16	6104	15.52	10094	15.29	26191	6547.75	14.83
	Cnidaria	1331	4.70	2577	6.00	2840	7.22	7683	11.64	14431	3607.75	8.17
	Arthropoda	2097	7.41	3294	7.67	2876	7.31	3668	5.56	11935	2983.75	6.76
	Chlorophyta	1751	6.19	2499	5.82	2025	5.15	3477	5.27	9752	2438	5.52
	Bacillariophyta	1144	4.04	2761	6.43	2316	5.89	1155	1.75	7376	1844	4.18
	Basidiomycota	1098	3.88	1560	3.63	1332	3.39	1910	2.89	5900	1475	3.34
	Nematoda	635	2.24	1121	2.61	951	2.42	1481	2.24	4188	1047	2.37
	Apicomplexa	568	2.01	948	2.21	789	2.01	1506	2.28	3811	952.75	2.16
	Echinodermata	266	0.94	412	0.96	413	1.05	526	0.80	1617	404.25	0.92
	Placozoa	230	0.81	331	0.77	355	0.90	333	0.50	1249	312.25	0.71
	Hemichordata	97	0.34	180	0.42	171	0.43	227	0.34	675	168.75	0.38
	Chytridiomycota	16	0.06	2	0.00	6	0.02	649	0.98	673	168.25	0.38
	Platyhelminthes	47	0.17	97	0.23	77	0.20	173	0.26	394	98.5	0.22
	Microsporidia	56	0.20	96	0.22	91	0.23	60	0.09	303	75.75	0.17
	unclassified (derived from Fungi)	0	0.00	4	0.01	111	0.28	95	0.14	210	52.5	0.12
	Phaeophyceae	4	0.01	28	0.07	32	0.08	96	0.15	160	40	0.09
	Euglenida	3	0.01	6	0.01	2	0.01	75	0.11	86	21.5	0.05
	Porifera	1	0.00	16	0.04	11	0.03	37	0.06	65	16.25	0.04
	Xanthophyceae	6	0.02	4	0.01	2	0.01	42	0.06	54	13.5	0.03
	Blastocladiomycota	1	0.00	13	0.03	12	0.03	24	0.04	50	12.5	0.03
	Glomeromycota	0	0.00	0	0.00	3	0.01	36	0.05	39	9.75	0.02
	Mollusca	0	0.00	4	0.01	2	0.01	6	0.01	12	3	0.01
	Annelida	0	0.00	1	0.00	1	0.00	3	0.00	5	1.25	0.00
	Bryozoa	0	0.00	1	0.00	0	0.00	3	0.00	4	1	0.00
	Chromerida	0	0.00	0	0.00	2	0.01	0	0.00	2	0.5	0.00
	Rotifera	0	0.00	0	0.00	0	0.00	1	0.00	1	0.25	0.00

Supplementary Table 2. Percent of hits that map to specific subsystems from the soil samples isolated from four different regions across the Kerala mangrove ecosystem.

Samples / Subsystems	VL1 4671371.3	PYN 4671370.3	MAL 4671369.3	PGD 4671368.3	Average	STD
Clustering-based subsystems	12.18	12.80	12.99	11.95	12.48	0.49
Carbohydrates	11.46	12.32	11.80	10.80	11.59	0.64
Amino Acids and Derivatives	9.53	9.83	10.17	9.55	9.77	0.30
Protein Metabolism	9.90	9.32	9.16	8.31	9.17	0.66
Miscellaneous	5.97	6.27	6.56	6.09	6.22	0.26
Respiration	6.10	5.55	5.01	4.99	5.42	0.53
Cofactors, Vitamins, Prosthetic Groups, Pigments	5.21	5.08	5.05	5.56	5.22	0.23
DNA Metabolism	4.50	4.34	4.48	4.44	4.44	0.07
RNA Metabolism	4.37	4.06	4.15	4.08	4.17	0.14
Cell Wall and Capsule	4.30	4.05	3.90	4.39	4.16	0.23
Membrane Transport	3.84	3.90	3.89	3.66	3.82	0.11
Virulence, Disease and Defense	3.37	3.38	3.13	3.74	3.40	0.25
Nucleosides and Nucleotides	3.28	3.18	3.16	2.86	3.12	0.18
Fatty Acids, Lipids, and Isoprenoids	2.18	2.41	2.49	2.59	2.42	0.17
Stress Response	2.21	2.32	2.61	2.37	2.38	0.17
Nitrogen Metabolism	1.80	1.72	1.60	2.85	1.99	0.58
Motility and Chemotaxis	1.83	1.19	1.16	2.45	1.66	0.61
Metabolism of Aromatic Compounds	0.99	1.26	1.64	1.71	1.40	0.34
Phosphorus Metabolism	1.20	1.28	1.29	1.28	1.26	0.04
Regulation and Cell signaling	1.16	1.05	1.12	1.30	1.16	0.10
Sulfur Metabolism	0.96	1.10	0.97	1.22	1.07	0.12
Phages, Prophages, Transposable elements, Plasmids	1.05	0.92	0.91	1.01	0.97	0.07
Cell Division and Cell Cycle	1.03	0.92	0.87	0.97	0.95	0.07
Potassium metabolism	0.62	0.59	0.65	0.81	0.67	0.10
Iron acquisition and metabolism	0.48	0.63	0.73	0.70	0.63	0.11
Secondary Metabolism	0.30	0.33	0.31	0.16	0.28	0.08
Dormancy and Sporulation	0.13	0.13	0.14	0.09	0.12	0.02
Photosynthesis	0.05	0.07	0.07	0.08	0.07	0.01

Supplementary Table 3. Functional analysis of single samples from each of four groups (India mangrove, Brazil mangrove, Puerto Rico forest, South China sea) using subsystem comparison showing the percentage or hits across all major groups.

Subsystem Level 1 / Samples	PGD	BrMgv2	PR Forest	E201-2	Average	Standard Dev	Z score
Carbohydrates	10.796	12.733	13.681	12.110	12.330	1.210	6.766
Clustering-based subsystems	11.953	12.283	12.183	10.652	11.768	0.757	6.204
Protein Metabolism	8.314	9.763	7.702	15.215	10.249	3.422	4.685
Amino Acids and Derivatives	9.554	9.944	9.043	10.780	9.830	0.733	4.266
Miscellaneous	6.089	6.397	6.746	5.194	6.106	0.665	0.542
Respiration	4.991	6.928	5.596	6.509	6.006	0.876	0.442
Cofactors, Vitamins, Prosthetic Groups, Pigments	5.555	5.352	4.797	5.150	5.213	0.323	-0.351
DNA Metabolism	4.437	4.567	3.956	4.900	4.465	0.391	-1.099
RNA Metabolism	4.078	4.085	3.468	5.827	4.365	1.017	-1.200
Membrane Transport	3.658	3.683	4.050	2.229	3.405	0.804	-2.159
Cell Wall and Capsule	4.394	3.633	3.513	1.806	3.336	1.092	-2.228
Nucleosides and Nucleotides	2.858	3.214	2.770	4.124	3.242	0.619	-2.323
Virulence, Disease and Defense	3.735	2.757	4.386	1.266	3.036	1.357	-2.528
Stress Response	2.367	2.350	2.696	2.625	2.510	0.177	-3.055
Fatty Acids, Lipids, and Isoprenoids	2.594	2.255	2.350	1.923	2.280	0.278	-3.284
Nitrogen Metabolism	2.848	1.355	1.556	2.406	2.041	0.705	-3.523
Metabolism of Aromatic Compounds	1.707	0.878	2.306	0.950	1.460	0.677	-4.104
Phages, Prophages, Transposable elements, Plasmids	1.007	0.973	1.673	1.338	1.248	0.328	-4.317
Phosphorus Metabolism	1.275	1.328	1.425	0.951	1.245	0.205	-4.320
Sulfur Metabolism	1.223	1.273	1.299	1.005	1.200	0.134	-4.364
Motility and Chemotaxis	2.455	0.746	0.845	0.382	1.107	0.920	-4.457
Cell Division and Cell Cycle	0.974	1.043	0.780	1.229	1.006	0.185	-4.558
Regulation and Cell signaling	1.296	0.857	1.265	0.590	1.002	0.340	-4.562
Potassium metabolism	0.809	0.464	0.900	0.110	0.571	0.360	-4.993
Iron acquisition and metabolism	0.695	0.509	0.549	0.209	0.490	0.204	-5.074
Secondary Metabolism	0.162	0.414	0.321	0.446	0.336	0.127	-5.228
Dormancy and Sporulation	0.094	0.138	0.075	0.060	0.092	0.034	-5.472
Photosynthesis	0.082	0.079	0.069	0.016	0.061	0.031	-5.503

Supplementary Table 4. Class level taxonomic distribution between and within across all the samples

Kingdom	Phylum		Kerala Mangrove						Brazil Mangrove						Puerto Rico Forest Sample	Ocean Sediment						Total Average	Total STD
		Sample Name	VL1	PYN	MAL	PGD	Average	STD	BrMgv1	BrMgv2	BrMgv3	BrMgv4	Average	STD	Puerto Rico	E201-1	E201-2	E208-1	E208-2	Average	STD		
		MG-RAST ID	4671371.3	4671370.3	4671369.3	4671368.3			4451033.3	4451034.3	4451035.3	4451036.3			4446153.3	4487294.3	4487295.3	4487376.3	4487377.3				
		**Total Bacteria reads**	3077852	3308800	3136206	3323940	3211699.50	123343.50	574258	102306	92059	91890	215128.25	239469.37	508545	819037	834223	765291	647811	766590.50	84528.20	1329401.4	1357656
		**Percent Bacteria of total**	95.42	96.96	97.37	97.47	96.81	0.95	96.89	95.07	96.22	95.09	95.82	0.89	97.70	85.72	85.33	92.04	91.18	88.57	3.53	94.035828	4.380521
		**Percent Bacteria of assigned**	95.42	96.96	97.37	97.48	96.81	0.95	96.89	95.07	96.22	95.09	95.82	0.89	97.71	85.72	85.33	92.04	91.19	88.57	3.53	94.038115	4.380064
Bacteria	***Proteobacteria***	***Gammaproteobacteria***	15.77	32.63	48.46	14.31	27.79	16.09	41.05	29.30	30.73	30.96	33.01	5.41	13.30	34.19	34.08	41.21	40.61	37.52	3.92	31.28	11.00
		Betaproteobacteria	23.80	19.20	14.04	68.59	31.41	25.11	15.71	15.04	17.45	16.47	16.17	1.04	21.05	12.86	12.70	14.17	13.80	13.38	0.72	20.38	14.86
		Alphaproteobacteria	8.94	20.07	22.92	11.56	15.87	6.68	21.10	19.94	23.13	22.47	21.66	1.42	52.18	43.60	43.82	28.82	29.02	36.31	8.54	26.74	12.74
		Deltaproteobacteria	23.83	21.49	13.52	5.09	15.98	8.50	20.16	33.26	26.14	28.14	26.93	5.41	12.75	8.16	8.20	14.19	15.17	11.43	3.77	17.70	8.57
		Epsilonproteobacteria	27.20	6.11	0.67	0.27	8.57	12.71	1.24	1.81	1.92	1.31	1.57	0.35	0.38	0.82	0.77	1.12	0.97	0.92	0.16	3.43	7.30
		unclassified	0.31	0.33	0.26	0.14	0.26	0.08	0.45	0.49	0.44	0.46	0.46	0.02	0.29	0.31	0.34	0.37	0.32	0.33	0.03	0.35	0.10
		Zetaproteobacteria	0.14	0.17	0.13	0.04	0.12	0.06	0.27	0.16	0.18	0.19	0.20	0.05	0.05	0.07	0.09	0.13	0.10	0.10	0.02	0.13	0.06
	Bacteriodes	***Bacteroidia***	31.93	13.83	12.35	16.92	18.76	8.99	36.27	37.23	30.83	32.93	34.31	2.97	19.69	22.72	22.50	27.20	25.65	24.52	2.29	25.39	8.24
		Cytophagia	17.64	13.44	16.74	25.12	18.23	4.93	13.67	14.94	15.90	15.76	15.07	1.02	26.06	18.06	18.63	15.01	16.95	17.16	1.60	17.53	3.91
		Flavobacteria	30.66	61.62	57.98	28.95	44.80	17.39	34.36	32.12	35.69	34.86	34.26	1.53	27.00	41.29	40.89	39.73	37.91	39.95	1.51	38.70	10.38
		Sphingobacteria	18.43	9.69	11.22	28.00	16.83	8.36	14.77	14.66	16.28	15.17	15.22	0.74	26.30	16.29	16.37	16.65	18.15	16.86	0.87	17.07	5.11
		unclassified (derived from Bacteroidetes)	1.34	1.42	1.72	1.02	1.37	0.29	0.93	1.05	1.30	1.28	1.14	0.18	0.95	1.65	1.61	1.41	1.33	1.50	0.15	1.31	0.26
	Firmicutes	***Bacilli***	27.22	33.08	36.63	41.67	34.65	6.08	40.98	29.90	32.70	29.65	33.31	5.30	40.03	45.17	44.29	45.67	41.71	44.21	1.76	37.59	6.41
		Clostridia	68.85	62.91	59.44	54.49	61.42	6.04	56.17	66.27	63.91	66.75	63.28	4.90	56.13	50.86	51.47	50.21	54.34	51.72	1.82	58.60	6.49
		Erysipelotrichi	0.74	0.84	0.85	0.87	0.83	0.06	0.52	0.82	0.61	0.75	0.68	0.13	0.59	0.95	1.07	0.91	0.90	0.96	0.08	0.80	0.16
		Negativicutes	3.19	3.17	3.09	2.96	3.10	0.10	2.32	3.00	2.77	2.85	2.74	0.29	3.25	3.01	3.18	3.22	3.05	3.11	0.10	3.00	0.25
	Actinobacteria	***Acidobacteria (class)***	8.73	6.53	6.99	7.82	7.52	0.97	4.21	4.02	4.46	4.56	4.31	0.25	12.77	4.68	5.23	4.05	4.06	4.51	0.56	6.01	2.57
		Solibacteres	22.03	17.59	15.46	12.59	16.92	3.98	11.60	10.58	11.06	13.01	11.56	1.05	22.61	8.99	10.07	6.17	6.44	7.92	1.92	12.94	5.22
		unclassified (derived from Acidobacteria)	11.65	7.97	7.87	9.68	9.29	1.78	6.67	5.71	5.79	6.44	6.15	0.47	14.96	6.66	6.74	5.89	5.81	6.28	0.49	7.84	2.76
		Actinobacteria (class)	57.58	67.91	69.68	69.92	66.27	5.86	77.52	79.69	78.69	75.99	77.97	1.59	49.67	79.67	77.95	83.88	83.69	81.30	2.96	73.22	10.15
Archaea		**Total Archaea reads**	115670	59600	43985	16934	59047.25	41660.69	13615	3946	2332	3469	5840.50	5227.03	6262	120209	126753	44972	45395	84332.25	45284.29	46395.538	42595.74
		**Percent Archaea of total**	3.59	1.75	1.37	0.50	1.80	1.30	2.30	3.67	2.44	3.59	3.00	0.73	1.20	12.58	12.97	5.41	6.39	9.34	3.99	4.4410309	4.188062
		**Percent Archaea of assigned**	3.59	1.75	1.37	0.50	1.80	1.30	2.30	3.67	2.44	3.59	3.00	0.73	1.20	12.58	12.97	5.41	6.39	9.34	3.99	4.4411582	4.188203
	Euryarchaeota	***Archaeoglobi***	7.57	8.02	7.06	5.18	6.96	1.25	8.27	8.55	9.63	8.51	8.74	0.60	5.77	5.19	8.81	7.88	8.43	7.58	1.64	7.61	1.41
		Halobacteria	6.22	12.37	13.98	13.40	11.49	3.58	10.13	8.12	10.09	7.98	9.08	1.19	18.42	14.93	15.63	12.16	13.56	14.07	1.54	12.08	3.45
		Methanobacteria	7.29	6.93	6.30	6.35	6.72	0.48	5.96	7.57	6.08	8.51	7.03	1.23	5.51	5.69	7.83	6.56	7.35	6.86	0.94	6.76	0.90
		Methanococci	9.59	10.54	10.95	7.55	9.66	1.52	10.81	12.17	11.53	11.49	11.50	0.56	8.11	12.49	8.65	9.72	9.39	10.06	1.68	10.23	1.55
		Methanomicrobia	45.54	39.06	40.09	44.75	42.36	3.26	42.27	40.17	38.67	40.55	40.42	1.48	42.73	36.83	38.43	44.49	42.32	40.52	3.51	41.22	2.70
		Methanopyri	1.62	1.36	1.37	1.25	1.40	0.16	2.10	1.76	1.54	2.28	1.92	0.33	1.17	1.48	0.93	0.43	0.84	0.92	0.43	1.40	0.50
		Thermococci	12.31	13.41	12.45	10.87	12.26	1.05	13.33	14.31	14.78	12.51	13.73	1.01	9.97	13.66	10.06	9.83	11.08	11.16	1.75	12.20	1.69
		Thermoplasmata	2.34	2.16	2.22	3.27	2.50	0.52	2.18	1.73	1.85	2.28	2.01	0.26	2.97	4.57	3.70	4.40	3.26	3.98	0.61	2.84	0.94
		unclassified (derived from Euryarchaeota)	7.50	6.13	5.56	7.37	6.64	0.95	4.95	5.61	5.82	5.89	5.57	0.43	5.37	5.16	5.97	4.51	3.77	4.85	0.94	5.66	1.02
	Crenarchaeota	***Thermoprotei***	100.00	100.00	100.00	100.00	100.00	0.00	100.00	100.00	100.00	100.00	100.00	0.00	100.00	100.00	100.00	100.00	100.00	100.00	0.00	100.00	0.00
	Thaumarchaeota	***unclassified (derived from Thaumarchaeota)***	100.00	100.00	100.00	100.00	100.00	0.00	100.00	100.00	100.00	100.00	100.00	0.00	100.00	100.00	100.00	100.00	100.00	100.00	0.00	100.00	0.00
Eukaryota		**Total Eukaryota reads**	28309	42955	39324	66029	44154.25	15857.13	4726	1329	1238	1252	2136.25	1726.96	5455	16004	16439	20596	16611	17412.50	2137.65	20020.538	20352.95
		**Percent Eukaryota of total**	0.88	1.26	1.22	1.94	1.32	0.44	0.80	1.23	1.29	1.30	1.16	0.24	1.05	1.68	1.68	2.48	2.34	2.04	0.42	1.4719236	0.455311
		**Percent Eukaryota of assigned**	0.88	1.26	1.22	1.94	1.32	0.44	0.80	1.24	1.29	1.30	1.16	0.24	1.05	1.68	1.68	2.48	2.34	2.04	0.42	1.4719644	0.455339
	Streptophyta	***Anthocerotopsida***	0.08	0.04	0.05	0.03	0.05	0.02	0.00	0.00	0.00	0.41	0.10	0.21	0.00	0.00	0.15	0.27	0.00	0.10	0.13	0.08	0.13
		Bryopsida	11.35	11.87	11.93	5.76	10.23	2.99	10.39	10.60	7.73	12.76	10.37	2.06	9.11	11.59	10.81	10.78	10.12	10.83	0.60	10.37	1.90
		Charophyceae	0.02	0.30	0.50	0.40	0.31	0.21	0.30	0.00	0.00	0.00	0.07	0.15	0.00	0.53	0.69	0.24	0.47	0.48	0.19	0.27	0.24
		Chlorokybophyceae	0.19	0.56	0.35	0.31	0.35	0.15	0.10	0.00	0.00	0.41	0.13	0.19	0.09	1.07	0.54	0.54	0.33	0.62	0.31	0.34	0.29
		Coleochaetophyceae	0.19	0.55	0.46	0.58	0.44	0.18	0.10	0.35	0.00	0.41	0.22	0.20	0.09	0.43	0.44	0.45	0.33	0.41	0.06	0.34	0.18
		Coniferopsida	0.10	0.17	0.13	0.07	0.12	0.04	0.10	0.71	0.43	0.82	0.51	0.32	0.00	0.53	0.33	0.33	0.29	0.37	0.11	0.31	0.26
		Cycadopsida	0.00	0.01	0.00	0.02	0.01	0.01	0.00	0.00	0.00	0.00	0.00	0.00	0.00	0.18	0.00	0.00	0.04	0.05	0.08	0.02	0.05
		Equisetopsida	0.00	0.00	0.00	0.01	0.00	0.00	0.00	0.00	0.00	0.00	0.00	0.00	0.00	0.00	0.00	0.00	0.04	0.01	0.02	0.00	0.01
		Gnetopsida	0.10	0.00	0.02	0.03	0.04	0.04	0.00	0.00	0.43	0.00	0.11	0.21	0.00	0.04	0.00	0.03	0.04	0.03	0.02	0.05	0.12
		Isoetopsida	5.66	6.06	5.70	2.59	5.00	1.62	4.35	3.18	3.43	4.12	3.77	0.55	4.90	5.62	5.30	5.03	4.75	5.17	0.37	4.67	1.08
		Jungermanniopsida	0.02	0.10	0.11	0.07	0.07	0.04	0.00	0.00	0.00	0.00	0.00	0.00	0.09	0.21	0.25	0.12	0.07	0.17	0.08	0.08	0.08
		Liliopsida	13.19	14.72	12.97	17.61	14.62	2.14	15.63	14.84	15.02	13.17	14.66	1.05	12.64	13.44	19.30	20.72	20.32	18.44	3.39	15.66	2.89
		Lycopodiopsida	0.02	0.00	0.02	0.00	0.01	0.01	0.00	0.00	0.00	0.00	0.00	0.00	0.00	0.07	0.07	0.09	0.11	0.09	0.02	0.03	0.04
		Marattiopsida	0.04	0.06	0.05	0.04	0.05	0.01	0.00	0.00	0.00	0.00	0.00	0.00	0.00	0.00	0.04	0.03	0.00	0.02	0.02	0.02	0.02
		Marchantiopsida	0.11	0.49	0.58	0.52	0.43	0.21	0.10	0.00	0.00	0.00	0.02	0.05	0.26	1.49	1.05	0.09	0.22	0.71	0.67	0.38	0.45
		Mesostigmatophyceae	0.31	0.30	0.57	0.29	0.37	0.13	0.00	0.00	0.43	0.41	0.21	0.24	0.09	0.39	0.65	0.51	0.29	0.46	0.16	0.33	0.20
		Polypodiopsida	0.15	0.13	0.06	0.03	0.09	0.06	0.00	0.00	0.00	0.00	0.00	0.00	0.00	0.07	0.07	0.09	0.18	0.10	0.05	0.06	0.06
		Zygnemophyceae	0.36	0.33	0.14	0.22	0.27	0.10	0.00	0.71	0.00	0.41	0.28	0.34	0.17	0.53	0.11	0.06	0.83	0.38	0.37	0.30	0.27
		unclassified	68.13	64.31	66.38	71.41	67.55	3.01	68.94	69.61	72.53	67.08	69.54	2.26	72.57	63.81	60.20	60.61	61.57	61.55	1.61	66.70	4.34
	Ascomycota	***Dothideomycetes***	3.76	4.72	3.97	9.25	5.42	2.58	6.75	3.57	2.67	3.89	4.22	1.76	5.48	3.64	3.89	3.58	4.55	3.92	0.44	4.59	1.73
		Eurotiomycetes	40.02	33.70	34.96	38.91	36.90	3.05	35.91	34.52	35.88	34.63	35.23	0.76	40.48	31.43	29.56	31.19	31.19	30.84	0.86	34.80	3.48
		Leotiomycetes	4.20	4.04	3.91	3.59	3.94	0.26	4.18	1.98	4.96	6.23	4.34	1.78	4.80	4.06	3.46	4.48	3.53	3.88	0.48	4.11	0.98
		Pezizomycetes	1.24	1.50	1.23	1.12	1.27	0.16	0.00	0.40	0.38	2.33	0.78	1.05	0.90	0.83	1.23	1.20	1.38	1.16	0.23	1.06	0.59
		Pneumocystidomycetes	0.02	0.01	0.01	0.00	0.01	0.01	0.00	0.00	0.00	0.00	0.00	0.00	0.00	0.00	0.03	0.03	0.00	0.01	0.02	0.01	0.01
		Saccharomycetes	22.78	26.30	25.60	19.83	23.63	2.95	23.79	24.60	25.95	22.57	24.23	1.42	9.80	27.15	28.60	31.02	30.70	29.37	1.83	24.52	5.45
		Schizosaccharomycetes	6.68	7.32	7.30	6.34	6.91	0.48	6.75	7.54	8.78	7.39	7.62	0.85	3.11	12.97	11.63	8.73	8.54	10.47	2.19	7.93	2.43
		Sordariomycetes	21.29	22.42	23.03	20.95	21.92	0.97	22.62	27.38	21.37	22.96	23.58	2.62	35.42	19.93	21.60	19.78	20.11	20.35	0.84	22.99	4.22
	Chordata	***Actinopterygii***	8.95	10.53	10.60	8.04	9.53	1.26	12.97	9.40	10.27	13.02	11.42	1.86	8.16	9.87	12.24	11.81	12.35	11.57	1.16	10.63	1.73
		Amphibia	20.18	11.76	11.01	36.08	19.76	11.65	5.68	9.83	9.38	12.50	9.34	2.81	14.44	6.14	6.77	7.12	6.96	6.75	0.43	12.14	8.25
		Appendicularia	0.02	0.02	0.00	0.03	0.02	0.01	0.00	0.00	0.00	0.00	0.00	0.00	0.00	0.04	0.00	0.03	0.00	0.02	0.02	0.01	0.01
		Ascidiacea	4.02	4.82	4.77	3.25	4.21	0.74	2.57	2.99	4.02	2.60	3.05	0.68	3.34	3.03	3.26	2.14	3.16	2.90	0.51	3.38	0.81
		Aves	5.92	8.13	6.94	4.24	6.31	1.65	3.78	7.26	4.91	2.60	4.64	1.99	5.08	6.14	7.68	5.50	6.48	6.45	0.91	5.74	1.61
		Chondrichthyes	0.00	0.00	0.02	0.00	0.00	0.01	0.00	0.00	0.00	0.00	0.00	0.00	0.00	0.00	0.07	0.00	0.04	0.03	0.04	0.01	0.02
		Mammalia	51.71	54.93	56.16	41.87	51.17	6.48	68.51	57.69	60.27	60.42	61.72	4.70	59.22	62.65	60.25	65.06	61.30	62.31	2.08	58.46	6.58
		Thaliacea	0.00	0.00	0.02	0.00	0.00	0.01	0.00	0.00	0.00	0.00	0.00	0.00	0.00	0.00	0.00	0.00	0.00	0.00	0.00	0.00	0.00
		unclassified (derived from Chordata)	9.19	9.81	10.48	6.50	9.00	1.75	6.49	12.82	11.16	8.85	9.83	2.76	9.76	12.13	9.74	8.34	9.72	9.98	1.58	9.61	1.86
	Arthropoda	***Arachnida***	9.97	8.11	7.89	8.18	8.54	0.96	7.89	8.51	9.52	5.88	7.95	1.54	7.69	8.27	9.50	6.89	5.49	7.54	1.73	7.98	1.32
		Branchiopoda	0.00	0.00	0.03	0.05	0.02	0.03	0.00	0.00	0.00	0.00	0.00	0.00	0.00	0.00	0.07	0.00	0.00	0.02	0.04	0.01	0.03
		Cephalocarida	0.00	0.00	0.00	0.00	0.00	0.00	0.00	0.00	0.00	0.00	0.00	0.00	0.00	0.00	0.00	0.00	0.08	0.02	0.04	0.01	0.02
		Diplopoda	0.00	0.00	0.00	0.00	0.00	0.00	0.00	0.00	0.00	0.00	0.00	0.00	0.00	0.08	0.00	0.12	0.08	0.07	0.05	0.02	0.04
		Diplura	0.00	0.00	0.00	0.00	0.00	0.00	0.00	0.00	0.00	0.00	0.00	0.00	0.00	0.08	0.07	0.00	0.00	0.04	0.04	0.01	0.03
		Ellipura	0.00	0.00	0.03	0.00	0.01	0.02	0.00	0.00	0.00	0.00	0.00	0.00	0.32	0.24	0.00	0.00	0.00	0.06	0.12	0.05	0.11
		Insecta	90.03	91.77	92.04	91.63	91.37	0.91	92.11	91.49	90.48	94.12	92.05	1.54	91.99	91.02	89.68	92.70	94.28	91.92	2.00	91.80	1.37
		Malacostraca	0.00	0.09	0.00	0.11	0.05	0.06	0.00	0.00	0.00	0.00	0.00	0.00	0.00	0.16	0.30	0.23	0.08	0.19	0.10	0.07	0.10
		Maxillopoda	0.00	0.03	0.00	0.03	0.01	0.02	0.00	0.00	0.00	0.00	0.00	0.00	0.00	0.08	0.15	0.06	0.00	0.07	0.06	0.03	0.05
		Merostomata	0.00	0.00	0.00	0.00	0.00	0.00	0.00	0.00	0.00	0.00	0.00	0.00	0.00	0.00	0.22	0.00	0.00	0.06	0.11	0.02	0.06
		Ostracoda	0.00	0.00	0.00	0.00	0.00	0.00	0.00	0.00	0.00	0.00	0.00	0.00	0.00	0.08	0.00	0.00	0.00	0.02	0.04	0.01	0.02
	Bacillariophyta	***Bacillariophyceae***	44.06	53.78	53.02	44.07	48.73	5.40	24.22	47.62	40.00	55.95	41.95	13.49	45.71	40.21	41.28	39.98	35.89	39.34	2.37	43.52	8.39
		Coscinodiscophyceae	55.94	45.85	46.24	54.81	50.71	5.41	75.78	52.38	60.00	44.05	58.05	13.49	54.29	58.74	56.94	60.02	64.01	59.93	3.00	56.08	8.44
		Fragilariophyceae	0.00	0.36	0.73	1.13	0.56	0.48	0.00	0.00	0.00	0.00	0.00	0.00	0.00	1.05	1.78	0.00	0.10	0.73	0.84	0.40	0.59
